# Improving the estimation of parameter uncertainty distributions in nonlinear mixed effects models using sampling importance resampling

**DOI:** 10.1007/s10928-016-9487-8

**Published:** 2016-10-11

**Authors:** Anne-Gaëlle Dosne, Martin Bergstrand, Kajsa Harling, Mats O. Karlsson

**Affiliations:** Department of Pharmaceutical Biosciences, Uppsala University, Box 591, 751 24 Uppsala, Sweden

**Keywords:** Sampling importance resampling, Parameter uncertainty, Confidence intervals, Asymptotic covariance matrix, Nonlinear mixed-effects models, Bootstrap

## Abstract

Taking parameter uncertainty into account is key to make drug development decisions such as testing whether trial endpoints meet defined criteria. Currently used methods for assessing parameter uncertainty in NLMEM have limitations, and there is a lack of diagnostics for when these limitations occur. In this work, a method based on sampling importance resampling (SIR) is proposed, which has the advantage of being free of distributional assumptions and does not require repeated parameter estimation. To perform SIR, a high number of parameter vectors are simulated from a given proposal uncertainty distribution. Their likelihood given the true uncertainty is then approximated by the ratio between the likelihood of the data given each vector and the likelihood of each vector given the proposal distribution, called the importance ratio. Non-parametric uncertainty distributions are obtained by resampling parameter vectors according to probabilities proportional to their importance ratios. Two simulation examples and three real data examples were used to define how SIR should be performed with NLMEM and to investigate the performance of the method. The simulation examples showed that SIR was able to recover the true parameter uncertainty. The real data examples showed that parameter 95 % confidence intervals (CI) obtained with SIR, the covariance matrix, bootstrap and log-likelihood profiling were generally in agreement when 95 % CI were symmetric. For parameters showing asymmetric 95 % CI, SIR 95 % CI provided a close agreement with log-likelihood profiling but often differed from bootstrap 95 % CI which had been shown to be suboptimal for the chosen examples. This work also provides guidance towards the SIR workflow, i.e.,which proposal distribution to choose and how many parameter vectors to sample when performing SIR, using diagnostics developed for this purpose. SIR is a promising approach for assessing parameter uncertainty as it is applicable in many situations where other methods for assessing parameter uncertainty fail, such as in the presence of small datasets, highly nonlinear models or meta-analysis.

**Electronic supplementary material:**

The online version of this article (doi:10.1007/s10928-016-9487-8) contains supplementary material, which is available to authorized users.

## Introduction

Nonlinear mixed effects models (NLMEM) are increasingly used to support drug development [1]. Even though NLMEM have been mainly employed for exploratory purposes, they have been advocated as powerful tools also in confirmatory settings [2]. In such settings, the adequacy of the structural and distributional assumptions inherent to NLMEM is particularly important in order to draw correct conclusions. One of the aspects requiring scrutiny is parameter uncertainty. Indeed, parameter uncertainty is key to make drug development decisions such as testing whether trial endpoints meet defined criteria, calculating the power of a prospected trial [3], taking a go/no go decision at an interim analysis [4], selecting doses for a Phase II trial [5], or optimizing dosing regimen [6]. Parameter uncertainty in NLMEM is usually obtained from the asymptotic covariance matrix at the maximum likelihood parameter estimates assuming a multivariate normal distribution, from a parametric or nonparametric bootstrap procedure, or less commonly from log-likelihood profiling.

Using the covariance matrix, model parameters are assumed to be normally distributed and their confidence intervals (CI) are computed from the standard errors obtained based on the inverse of the observed Fisher information matrix [7]. However, when the settings are far from asymptotic conditions (i.e., when the number of observations and/or individuals is low) or when models display substantial nonlinearity, the assumption of normality may not hold. Reparameterization, for example using exponentiated parameters, has been advocated as an approach to address non-normal uncertainty [8]. However, the adequacy of the new distribution is by no means guaranteed. Determining whether the covariance matrix is appropriate, i.e., quantifying the distance from asymptotic conditions or the degrees of nonlinearity, is not straightforward. Measures of nonlinearity have been proposed [9] but they are controversial [10] and have not been applied to NLMEM. In addition, numerical issues may hamper the computation of the inverse of the FIM [11] and prevent its use.

The bootstrap [12] enables parametric or nonparametric parameter CI to be computed from a given number of parameter vectors which are estimated based on bootstrapped datasets. Nonparametric case bootstrap methods, where no assumption about uncertainty distribution is made, are the most commonly used in NLMEM. In nonparametric case bootstrap, new datasets are computed by resampling with replacement individual data from the original dataset. The new datasets are of the same size as the original dataset, but differ in the included individuals. The authors refer to Thai et al. [13] for a comparison of the performance of different bootstrap methods in NLMEM. Usage of the bootstrap may be limited due to time as it requires a high number of potentially computationally intensive estimation steps, even though some efforts have been made to reduce this [14]. A further limitation is that it is not applicable when data from only few individuals is available, when frequentist priors are used (a parameter for which uncertainty is high because of uninformative data would appear precise as all of its bootstrapped values would stay close to its prior value) or when doing model-based meta-analysis (different studies are typically not exchangeable). There is also a lack of consensus about both the handling of bootstrapped samples for which the estimation was not successful, and about which method to use to compute bootstrap CI. Just as for the covariance matrix, it is difficult to judge in which situations the use of bootstrap is appropriate. Recent work suggests that datasets commonly used in NLMEM are actually too small for the bootstrap to be properly applied [13, 15].

Log-likelihood profiling [16, 17] can also be used to assess parameter uncertainty. Like the bootstrap, this method makes no assumption about uncertainty distribution. Parameter CI are computed in a univariate manner by estimating the objective function value (OFV), which corresponds to minus two times the log-likelihood up to a constant, at an array of fixed values of the parameter of interest while the other parameters are estimated. Values which lead to differences in OFV (dOFVs, calculated as the OFV at a given array of parameter estimates minus the OFV at the maximum likelihood estimates) equal to the value of the Chi square distribution for one degree of freedom and at the α confidence level are taken as the outer bounds of the 1 − α % CI. Minimization problems and long runtimes can be an issue, even if the number of estimations needed is typically much lower than with the bootstrap. The main drawback of log-likelihood profiling is that it does not provide full uncertainty distributions. Only the bounds of a parameter’s CI are available, and despite some work on multivariate implementation [18], as of now it cannot be used to generate entire sets of parameters.

All currently available methods for assessing parameter uncertainty thus present non negligible drawbacks in NLMEM settings. Sampling importance resampling (SIR), an alternative method making no distributional assumptions on uncertainty and devoid of repeated estimation steps, could address some of these shortcomings.

The SIR algorithm has been proposed by Rubin [19] to obtain posterior parameter uncertainty distributions. SIR was developed in the Bayesian setting as a noniterative and universally applicable method for obtaining draws from an unknown distribution based on draws from an approximation of this distribution. The draws are resampled based on their importance ratios, which measure the agreement between the approximated distribution and the data at hand, and are expected to be proportional to the resampling probabilities given the unknown distribution. Starting from any proposal distribution, SIR will thus resample a set of samples representative of the unknown distribution. In the past, SIR has been used to calculate marginal densities [20], to impute missing data [21] and in Bayesian modelling [22, 23]. It has been applied in the healthcare domain to project trends in HIV/AIDS epidemics [24], to estimate cost-effectiveness [25] and to perform optimal experimental design in systems pharmacology [26]. However, to the authors’ knowledge the application of SIR to estimate parameter uncertainty distributions in NLMEM has not previously been described in the literature.

The aim of this work was to develop a workflow for performing SIR in the context of NLMEM, and to apply this workflow to compare parameter uncertainty obtained with SIR to parameter uncertainty obtained with commonly used methods.

## Methods

### SIR algorithm and implementation in NLMEM

The objective of SIR is to provide, for a given model and a given set of data, a set of *m* parameter vectors which are representative of the true and unknown parameter uncertainty distribution. SIR is performed in the following three steps:

1. Step 1 (sampling): *M* (*M* > *m*) parameter vectors are randomly sampled from a proposal distribution.
2. Step 2 (importance weighting): For each of the *M* sampled parameter vectors, an importance ratio is computed. This importance ratio corresponds to the probability of being sampled in the true parameter uncertainty distribution. It is computed as the likelihood of the data given the parameter vector, weighted by the likelihood of the parameter vector in the proposal distribution (Eq. 1).

1 $$IR = \frac{{\exp \left( {\frac{ - 1}{2}dOFV} \right)}}{relPDF},$$

where IR is the importance ratio, dOFV is the difference between the objective function value (OFV) of the parameter vector and the OFV of the final parameter estimates on the original data, and *relPDF* is the value of the probability density function of the parameter vector relative to the probability density of the final parameter estimates.

(3) Step 3 (resampling): In the last step, *m* parameter vectors are resampled from the pool of *M* simulated vectors based on their importance ratio. These vectors can be used to compute desired CI.

**Fig. 1:**
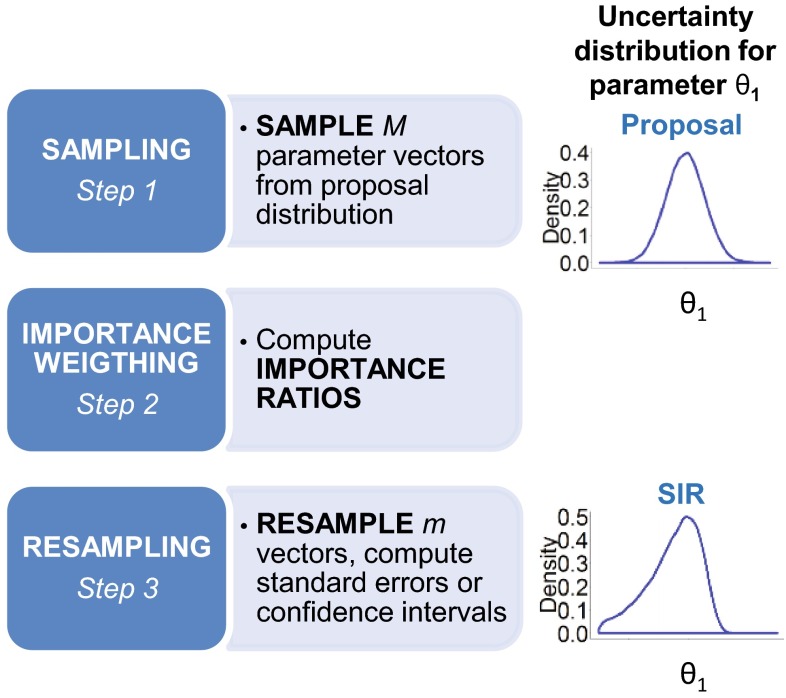
The three steps of the SIR procedure

A summary of the SIR procedure is provided in Fig. 1. Full details on the SIR rationale and implementation are provided in Online Resource 1.

### SIR workflow

In theory, SIR results should be independent on the chosen SIR settings, which are the proposal distribution and the number of samples *M*. However this is not always the case, for example if the proposal is very far from the true distribution and the number of samples is too low. In this work, settings which would be in general appropriate for NLMEM were explored, and diagnostics to judge a posteriori whether SIR settings should be improved were developed.

SIR was initially proposed to be performed based on a default workflow, where the estimated “sandwich” variance–covariance matrix, which is a function of the Hessian and the cross-product gradient, was used as proposal distribution. The number of samples *M* was set to 5000 and the number of resamples *m* was set to 1000.

Potential improvements of this workflow were then investigated. First, different numbers of samples were investigated (*M* = 2000, 4000, 6000, 8000 and 10,000). The number of resamples *m* was not modified, as this number was chosen in order to have sufficient precision on the outer bounds of the CI of interest, which was the 95 % CI. Note that *m* can be chosen freely depending on the desired precision of the uncertainty. What is important for SIR is thus not so much the intrinsic value of *M*, but rather its relation to *m*, expressed as *M/m* ratios (*M/m* = 2, 4, 6, 8 and 10). One should choose *m* in the same manner as the number of samples in a bootstrap, i.e., depending on the intended use. For example, *m* = 1000 would be recommended to compute a 95 % CI. *M* would then derived from the recommended *M/m* ratio, 5 by default, to be 5000. Secondly, different proposal distributions were investigated. They corresponded to inflations and deflations of the covariance matrix, for which all variances and covariances of the uncertainty distribution were either increased or decreased by a certain factor (0.5, 0.75 1.5 and 2). It is very important to note that proposal distributions not based on the covariance matrix can also be used, especially if the covariance matrix is not estimable. This is a major advantage of SIR which will be further detailed in the discussion. Lastly, performing SIR using resampling with replacement in order to increase SIR efficiency was also investigated. Because replacement was not recommended, results from this investigation are not discussed here, but they are provided in Online Resource 2.

### Application of SIR to simulated examples and real data examples

SIR was used to obtain parameter uncertainty of two simulation examples and three real data examples. For the real data examples, 95 % CI of model parameters were compared to the CI obtained from the covariance matrix, bootstrap (1000 samples, no stratification) and log-likelihood profiling. For the simulation examples, 95 % CI were also computed and used to calculate the proportion of datasets for which the computed CI included the true simulation value, a metric known as coverage. The coverage obtained with SIR was compared to the coverage obtained with the covariance matrix. As uncertainty depends on the estimation method, all comparisons should be done using the same method. The first order conditional estimation with interaction method was used in this work.

The three real data examples were moxonidine [27], pefloxacin [28] and phenobarbital [29]. Models were multiple dose pharmacokinetic (PK) 1-compartment models with oral or intravenous (i.v.) administration. Data ranged from rich to sparse. The simulation models comprised an i.v. 1-compartment PK model with first-order elimination and a pharmacodynamic (PD) dose–response Emax model. Simulated datasets comprised 20, 50 or 200 individuals, each providing four observations. For each model and dataset size, 500 simulations and re-estimations were performed. Details on model structures, parameters and designs are available in Online Resource 3.

### SIR diagnostics

**Fig. 2:**
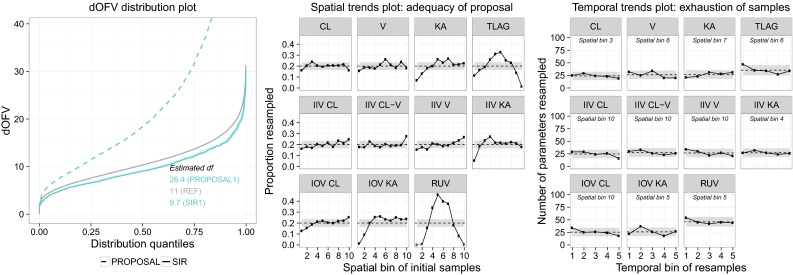
Diagnostic plots for the moxonidine real data example showing that SIR settings were appropriate. The SIR dOFV distribution is below the Chi square distribution in the dOFV distribution plot (*left panel*). Diagonal upward and bell-shaped trends in the spatial trends plot (*middle panel*) indicate that the proposal distribution is different from the true uncertainty. Horizontal trends in the temporal trends plot (*right panel*) show that the *M/m* ratio was high enough for SIR to compensate the inadequacy of the proposal distribution. See text in the “Methods” section and additional legends in Online Resource 4 for further details. (*CL* clearance, *V* volume, *KA* absorption rate, *TLAG* lag-time, *IIV* inter-individual variability, *IOV* inter-occasion variability, *RUV* residual unexplained variability)

When performing SIR, the user needs to choose a proposal distribution and a samples/resamples ratio *M/m*. Diagnostics were developed to judge whether the chosen SIR settings were appropriate, i.e., whether SIR results could be considered final. Three graphical diagnostics were developed: (1) the dOFV distribution (first proposed in [15] in connection with bootstrap), which is a global diagnostic assessing the adequacy of both the proposal distribution and *M/m* for all parameters simultaneously (2) the spatial trends plot, which is a local diagnostic assessing the adequacy of the proposal distribution separately for each parameter and (3) the temporal trends plot, which is a local diagnostic assessing the adequacy of *M/m* separately for each parameter. An illustration of the diagnostics is available in Fig. 2. The diagnostics should be used as follows: SIR settings are considered adequate if the dOFV distribution after SIR is at or below the Chi square distribution and no trends are apparent in the temporal trends plot. The investigations on the SIR workflow will provide guidance towards what to do when this is not the case.

#### dOFV distribution plot (left panel, Fig. 2)

dOFV quantile distributions were suggested as a method to diagnose the appropriateness of bootstrap uncertainty distributions [15] and were applied to SIR. The principle behind this diagnostic is that if the parameter vectors resampled by SIR were the true uncertainty, the difference between their OFV and the OFV obtained at the final parameter estimates of the model should follow a Chi square distribution. The degrees of freedom (df) of this distribution is equal to the number of estimated parameters for an unconstrained fixed effects model [30], but the exact df is unknown for NLMEM. Indeed, it is expected to be equal to or below the number of estimated parameters, notably due to the estimation of random effects and other bounded parameters, which may not account for full degrees of freedom [31]. Plotting the dOFV distributions obtained from the *M* proposal samples and from the *m* SIR resamples against a Chi square distribution with degrees of freedom equal to the number of estimated parameters informs about the adequacy of the proposal distribution and *M/m*. If the dOFV distribution obtained from the *M* samples is at or close to the Chi square distribution, it means that the proposal distribution is close to the true distribution; if it is far above or below, it means that it is quite different from the true distribution. If the dOFV distribution obtained from the *m* SIR resamples is at or below the Chi square distribution, *M/m* may have been sufficient and should be further investigated with the temporal trends plot; if the distribution is above the Chi square distribution, *M/m* was not sufficient. The mean of the dOFV distribution can be used to quantitatively compare the distributions; it corresponds to the degrees of freedom if the distribution is a Chi square distribution.

#### Spatial trends plot (middle panel, Fig. 2)

This plot enables to visualize, parameter by parameter, whether the proposal distribution differs from the true uncertainty. The spatial trends plot shows the resampling proportion, i.e., the number of resampled parameters divided by the number of parameters available from the *M* samples (on the y-axis), in different regions, or bins, of the parameter space (on the x-axis). The parameter space is defined as all values comprised between the lowest sampled parameter value and the highest sampled parameter value, and is divided into ten bins which all contain an equal number of samples. Four types of trends can be observed in this plot:

- *Horizontal trend* (i.e., *no trend*): If the observed resampling proportion is within stochastic noise around the expected proportion for all bins, it means that the proposal distribution is close to the true uncertainty.
- *Bell*-*shaped trend*: If the observed proportion is higher in the center and lower at the ends, it means that parameters close to the final estimates are resampled more often than those further away from them, and thus that the proposal distribution is wider than the true distribution.
- *u*-*shaped trend*: Oppositely, if the observed proportion is lower in the center and higher at the ends, it means that the proposal distribution is narrower than the true distribution.
- *Diagonal trend*: If the proportion is higher at one end and lower at the other, it means that the proposal distribution has a different (a)symmetry than the true distribution.

The spatial trends plot indicates how the proposal differs from the true distribution, but it does not inform whether SIR was able to compensate for these differences.

#### Temporal trends plot (right panel, Fig. 2)

This plot indicates, parameter by parameter, whether *M/m* was high enough to compensate for the differences between the proposal distribution and the true uncertainty. SIR can succeed as long as there are enough “good” samples available in the proposal distribution. The limiting factor for this is the bin that shows the highest proportion of resamples in the spatial trends plot. This bin, referred to as the top spatial bin, is the region of the parameter space where SIR is most likely to run out of “good” samples if *M/m* is not sufficient. Instead of binning sampled parameters based on their value as for the spatial trends plot, resampled parameters are now binned based on the order in which they were resampled: the parameters that were resampled first (i.e., most likely those with the highest importance ratios) belong to the first “time bin”, and those that were resampled last belong to the last bin. The temporal trends plot then shows, for each of the 5 time bins (on the x-axis), the number of resamples that belongs to the top spatial bin (on the y-axis) together with the sampling noise. Two trends can be observed for this diagnostic:

- *Horizontal trend* (i.e., *no trend*): If the number of resampled parameters in the top spatial bin is within sampling noise for all time bins, *M/m* was sufficient to compensate for potential differences between the proposal distribution and the true uncertainty.
- *Downward diagonal trend:* If on the other hand the number of resampled parameters decreases over time, it indicates a depletion of samples in the top bin and that there were not enough good samples in the SIR procedure to fully correct the proposal uncertainty: *M/m* was not sufficient.

### Software

NONMEM 7.2 and 7.3 [32] aided by PsN 3.5.9 and above were used for data simulation, model fitting and SIR computation. RStudio 0.98 using R 3.1.2 [33] was used for SIR computation and graphical output.

## Results

SIR was implemented as a modelling supporting tool in the PsN software and is thoroughly documented in the PsN user guide. It is fully automated and enables the user to easily perform SIR with any NONMEM-based model, providing numerical summary results such as relative standard errors (RSE) and CI as well as extensive graphical diagnostics.

### Real data examples: SIR diagnostics and comparative parameter CI 95 %

SIR settings with the default workflow proved appropriate based on the developed diagnostics for the three real data examples. In all examples, the uncertainty given by the covariance matrix appeared different from the true distribution, as evidenced by dOFV distributions above the Chi square distribution and non-horizontal trends in the spatial trends plots. A *M/m* ratio of 5 was sufficient to compensate the inadequacy in all examples: the dOFV distributions of the SIR resamples were below the Chi square distribution and the temporal trends plots displayed no trends.

For the moxonidine example (Fig. 2), the dOFV distribution of the proposal was well above the Chi square distribution (df 26.4 vs. 11), indicating that the asymptotic covariance matrix was relatively far from the true uncertainty. This was confirmed by the spatial trends plot, which showed bell-shaped trends for lag-time (TLAG), absorption rate (KA), residual unexplained variability (RUV) as well as diagonal trends for inter-individual variability on volume (IIV V) and inter-occasion variabilities (IOVs), evidencing an overestimation of parameter uncertainty and a misspecification of the symmetry, respectively. The dOFV distribution of the resamples, i.e., after SIR, was much improved over that of the proposal and was below the Chi square distribution (df 9.7). To check whether these results could be considered final, the temporal trends plot was inspected: it showed limited downward trends, mostly within sampling noise. To make sure that *M/m* had been sufficient to fully correct the proposal, SIR was performed with an additional 5000 samples from the covariance matrix (*M/m* = 10): the df of the resamples dOFV distribution did not change (df 9.6 vs. 9.7 previously), nor did parameter CI. SIR results with *M* = 5000 were thus considered final. For pefloxacin (Fig. A3 panel a, Online Resource 4), the dOFV distribution of the proposal was also above the Chi square distribution (df 14.2 vs. 10). The spatial trends plot showed an overprediction of the uncertainty for some parameters (RUV and covariate effect on clearance CL) and some discrepancy in symmetry (IIV V and IOV V). The dOFV distribution after resampling was below the Chi square distribution (df 8) and no trends were apparent in the temporal trends plot, suggesting that the chosen *M/m* of 5 was sufficient. For phenobarbital (Fig. A3 panel b, Online Resource 4), the dOFV distribution of the proposal was only slightly above the Chi square distribution (df 9 vs. 7), showing good adequacy overall. Diagonal upward trends indicating asymmetry in the true variance uncertainties were present in the spatial trends plot. As the dOFV resamples distribution was 0.6 df lower than the Chi square and the temporal trends plot showed no trend, SIR results were considered final.

**Fig. 3:**
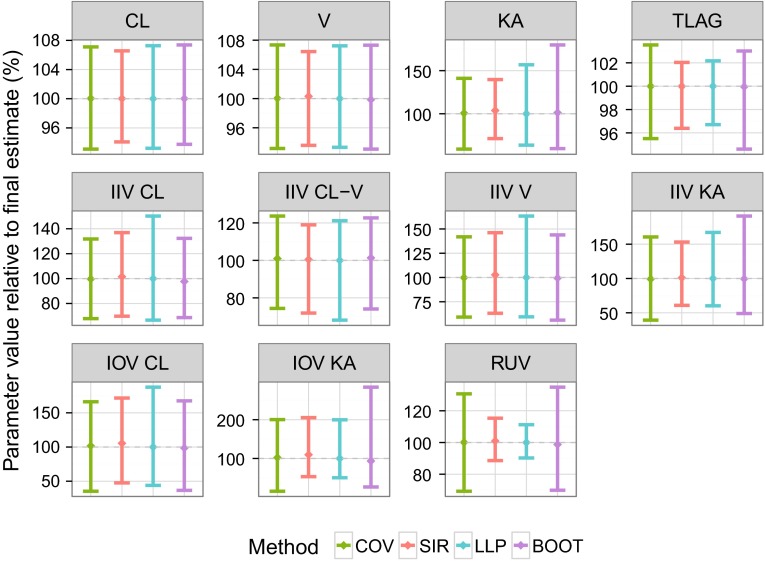
Comparative 95 % CI of the moxonidine model parameters between four uncertainty methods: covariance matrix (*COV, green*), sampling importance resampling (*SIR, red*), log-likelihood profiling (*LLP, blue*) and bootstrap (*BOOT, violet*). Vertical *error bars* represent the 95 % CI and the points represent the median of the uncertainty distributions, expressed relative to the final parameter estimate. All random effects are analyzed on the variance scale

Once SIR settings were proven appropriate, SIR parameter 95 % CI (using *M* = 5000 for all models) could be compared to those obtained from the covariance matrix, bootstrap and log-likelihood profiling. For moxonidine (Fig. 3), all methods provided similar 95 % CI for CL and V. IIV estimates of CL and V were similar across methods except for log-likelihood profiling, for which upper bounds were increased. KA and TLAG 95 % CI were similar for SIR and log-likelihood profiling, narrower than those obtained with the covariance matrix and displaying asymmetry. Asymmetry in the uncertainty of absorption parameters was even more marked when using bootstrap. Regarding IOVs, SIR, log-likelihood profiling and bootstrap led to high asymmetry especially for IOV KA. Uncertainty in RUV was symmetric but narrower with SIR and log-likelihood profiling than with the covariance matrix or bootstrap. Similar observations were made with the pefloxacin and phenobarbital examples (Fig. A4 panel a, b, Online Resource 5). In terms of runtime the covariance matrix was the fastest method, followed by log-likelihood profiling, SIR and finally bootstrap (namely 14 s, 15 min, 1 and 2 h respectively for the moxonidine example; relative differences were similar for the other examples).

### Simulation study: comparative coverage

**Fig. 4:**
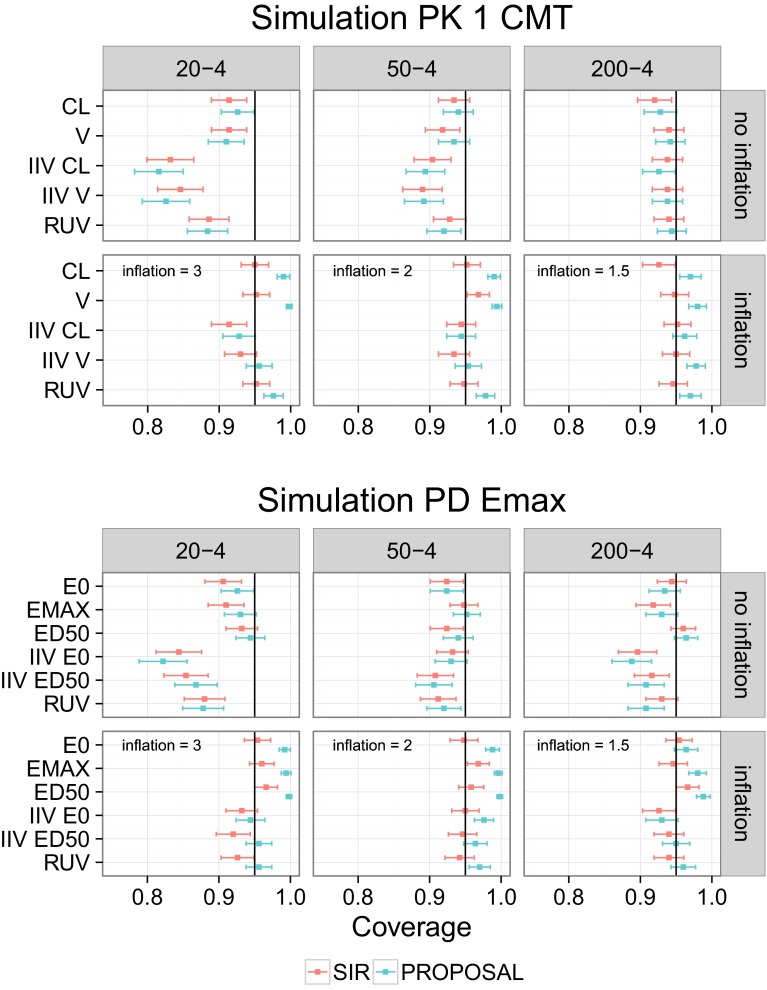
Coverage with SIR is as good as or better than coverage with the covariance matrix. The *squares* represent the observed 95 % coverage for the parameters of the two simulation examples with SIR (*red*) and with the proposal distribution (*blue*). The horizontal *error bars* represent the 95 % CI around the observed coverage (500 simulated datasets per example and dataset size). SIR was performed both with the default workflow (“no inflation” panels: covariance matrix as proposal distribution and *M/m* = 5) and with an optimized workflow (“inflation” panels: covariance matrix inflated by 3, 2 and 1.5 for the datasets with 20, 50 and 200 individuals respectively as proposal distributions and *M/m* = 5)

Coverage with SIR was similar to coverage with the covariance matrix when using the latter as proposal distribution. SIR results could however be improved when inflating the proposal distribution (Fig. 4).

In both simulation examples, coverage with the covariance matrix improved with increasing sample size. 95 % CI often underestimated the true parameter uncertainty. Most parameters displayed suboptimal coverage at low sample size; coverage was worse for IIV parameters, around 0.85 instead of 0.95. Coverage was however satisfactory at high sample size, except for IIVs in the PD example which had a coverage of 0.90. SIR results without inflation were similar to or slightly improved over the covariance matrix, but from the diagnostic plots it was apparent that SIR settings were not fully appropriate. In most cases, the dOFV distribution of the proposal was below the Chi square distribution and diagonal trends were apparent in the temporal trends plots. Based on the investigations on the SIR workflow presented in the next paragraph, SIR was performed again using inflations of the covariance matrix as proposal distributions. Inflation factors were chosen as the lowest value (starting from 1 and increasing by steps of 0.5) for which diagnostic plots looked appropriate. The inflation factor was 3 for datasets with 20 individuals, 2 for datasets with 50 individuals, and 1.5 for datasets with 200 individuals. SIR coverage was greatly improved: only IIV CL and IIV ED50 in datasets with 20 individuals were still statistically suboptimal using the optimized SIR workflow.

### Impact of *M/m* and proposal distribution on SIR results

Changes in SIR settings, i.e., the *M/m* ratio and the proposal distribution, were investigated to better understand their impact on SIR results so as to give guidance on how to choose SIR settings. The investigations, based on the real data examples, showed that the *M/m* ratio necessary for SIR results to be considered final was different in the three investigated examples, and was lower the closer the proposal distribution was to the Chi square distribution. The proposal distribution was found to have a profound impact on SIR results: distributions slightly wider than the true distribution performed best, while distributions narrower than the true distribution performed badly. The diagnostic plots were able to distinguish between appropriate and inappropriate settings in all cases.

#### M/m ratio

**Fig. 5:**
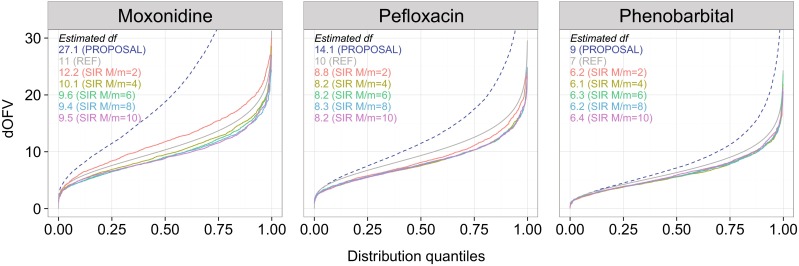
SIR dOFV distributions converge with increasing number of initial samples. Comparative dOFV distributions and estimated df for the covariance matrix as proposal distribution (*blue dotted line*), SIR with increasing number of initial samples (*colored full lines*) and the reference Chi square (*grey full line*) for the three real data examples

The optimal *M/m* ratio was found to be 6 for moxonidine, 4 for pefloxacin and 2 for phenobarbital, as evidenced by the visual stabilization of the dOFV distributions at *M/m* = 6, *M/m* = 4 and *M/m* = 2 respectively (Fig. 5). The df was very stable for ratios above the optimal ratio, with variations around 0.1–0.2 df.

The correspondence between the visual stabilization of the dOFV distributions and the numerical stabilization of RSE and CI bounds, which are the real metrics of interest for parameter uncertainty, was investigated. It was found that visually stable dOFV distributions corresponded to relatively stable RSE and CI bounds, and vice versa. In the case of moxonidine for example, parameter RSE using an *M/m* ratio of 10 instead of 8 changed on average by less than 1.9 % of their value (range [0.1–5.2 %], see Online Resource 6). How fast RSE and CI stabilized over increasing *M/m* differed greatly between parameters. The RSE and CI bounds appeared quite stable from the lowest *M/m* ratio for CL, V and IIV V, whereas for KA, TLAG, IIV CL and IOV CL stabilization did not occur until *M/m* = 4. Stabilization was slowest for IIV KA and IOV KA, occurring around *M/m* = 6.

#### Proposal distribution

**Fig. 6:**
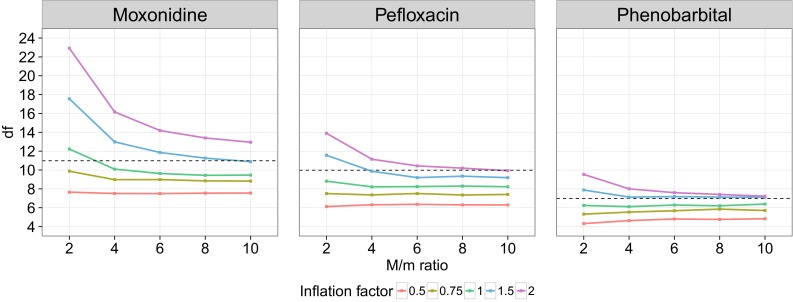
Degrees of freedom of the SIR dOFV distributions do not fully converge between inflation factors over increasing number of samples. *Squares* represent the df for SIR with increasing inflation factors (*colored full lines*) as a function of the number of samples *M* for the three real data examples. The *horizontal dashed lines* correspond to the number of estimated parameters for each model

The impact of the proposal distribution was investigated to see whether SIR would lead to better results when using modifications of the covariance matrix as proposal distributions. In this work only modifications of the covariance matrix were investigated, but it is important to stress that any distribution can be used as proposal distribution for SIR and that the covariance matrix is thus not required for SIR to be performed. Figure 6 displays the estimated df of the SIR dOFV distributions as a function of *M/m* for different proposal distributions. As expected, the proposal distributions affected how fast SIR stabilized: higher *M/m* ratios were necessary to reach stable dOFV distributions when starting from inflations of the covariance matrix. Stabilization of the df was not reached for any of the models at an *M*/*m* ratio of 10 when starting from a twofold inflation of the covariance matrix, and was also not reached at *M*/*m* = 10 for the 1.5-fold inflation of the moxonidine covariance matrix. For deflations of the covariance matrix on the other hand, stabilization seemed faster, even if slow degrees of freedom increases were visible, in particular for phenobarbital. However, the diagnostics plots when using deflated proposal distributions showed trends indicating too low *M/m* ratios in the temporal trends plots. A stable df was thus not a good indicator of SIR performance when the proposal distribution was too narrow (i.e., below the Chi square distribution). Given that the degrees of freedom was still much lower for the deflations than for the inflations at *M/m* = 10, it appeared that too narrow proposal distributions would need much higher ratios to converge than too wide proposal distributions. Lastly, the degrees of freedom obtained with SIR starting from the deflations and inflations of the covariance matrix did not converge at the highest tested *M/m* ratio of 10. Differences in df at this point spanned 5 df for moxonidine, 4 df for pefloxacin and 2 df for phenobarbital.

## Discussion

SIR was successfully implemented in the NLMEM framework and is available as a user-friendly modelling support tool in the PsN software. SIR was able to characterize parameter uncertainty more accurately than conventional methods such as the covariance matrix, the bootstrap or log-likelihood profiling. A workflow based on specifically designed diagnostics was proposed to guide the user on how to perform SIR.

### SIR workflow

**Fig. 7:**
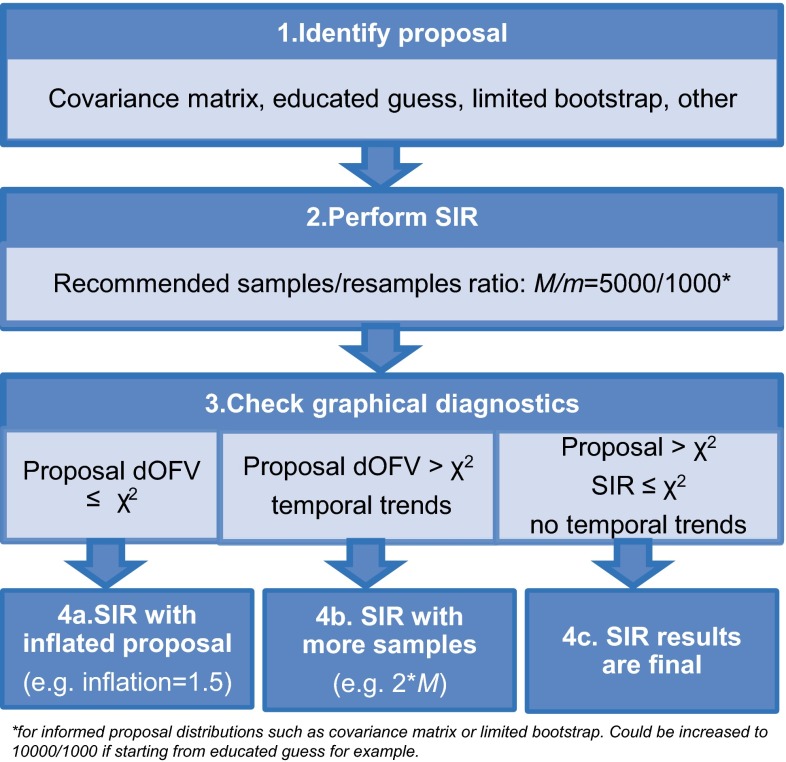
Decision tree for how to perform SIR in practice. χ^2^ is the Chi square distribution

Based on the performed investigations, the following decision tree (Fig. 7) is recommended when performing SIR:

1. Choose the best guess of the uncertainty distribution. The covariance matrix can be used if available, but it is not necessary: a limited number of bootstrap samples, or any other guess of the uncertainty distribution can be used as proposal distribution.
2. Perform SIR. The recommended samples/resample ratio *M/m* is 5 (for proposal distributions expected to be close to the true uncertainty, such as the covariance matrix), but may be increased to 10 for example (for other, less well informed proposal distributions).
3. Check the diagnostics. If the proposal dOFV distribution is below the Chi square distribution (u-shaped trends will be present in the spatial trends plot), restart SIR with a wider proposal distribution (for example using an inflation factor of 1.5). If the proposal dOFV distribution is above the Chi square distribution and trends are apparent in the temporal trends plot, increase *M* (double for example). If the proposal dOFV distribution is above the Chi square, no trends are apparent in the temporal trends plot and the SIR dOFV distribution is below the Chi square distribution, SIR results can be considered final.

### Real data examples: comparison of SIR with other methods for parameter uncertainty

Interpretation of differences in parameter 95 % CI between methods is not straightforward with real data examples as the true uncertainty remains unknown. Although some case studies are available [34], thorough comparisons of the performance of available methods to assess parameter uncertainty such as in Donaldson et al. [35] for nonlinear fixed effects models are lacking in NLMEM. In the investigated real data examples, starting from symmetric covariance matrix-based uncertainty, SIR was able to pick up expected asymmetry for given parameters such as variances and nonlinear processes (Figs. 3, A4, Online Resource 5). Results were often closest to those obtained with log-likelihood profiling, which could be expected as both rely on evaluation of the log-likelihood on the original dataset. However, contrarily to SIR, uncertainty using log-likelihood profiling is univariate and cannot be used for simulation. In addition, log-likelihood profiling requires reparameterization of the models featuring correlations between variances, since single matrices elements cannot be fixed in NONMEM. Bootstrap results also displayed the presence of asymmetry in the uncertainty distribution of selected parameters, but bootstrap CI were in general slightly wider than with SIR. However, bootstrap behaviour had been shown to be suboptimal for the considered real data examples [15]. In addition, in the pefloxacin example half of the bootstrap samples were unsuccessfully minimized, further evidencing the complications of this method to assess parameter uncertainty as the way to handle failed runs is not standardized and leads to different results. Contrarily, with SIR the likelihood of the data need only be evaluated, not estimated, which avoids issues related to convergence problems. Furthermore, as SIR evaluates likelihoods over the entire parameter space defined by the proposal distribution, it might help detecting if the final estimates were in a local minimum. A warning is printed if the SIR procedure detects one or more parameter vector(s) leading to lower OFV than the final estimates, which can help the user detect local minima. Both the performance of the default SIR workflow and the observed differences in 95 % CI between uncertainty methods in these real data settings are dependent on the chosen real data examples, which were all PK models with 1-compartment first order elimination. While further research is ongoing to identify potential limitations of SIR, there is little reason to believe that the results obtained here are not generalizable to other, more complex models.

Regarding runtime, SIR is expected to provide significant runtime gains over bootstrap, but quantification of this gain is not easily generalizable from the investigated examples. Runtime gains depend on two aspects: the adequacy of the proposal density, which determines the necessary *M/m* ratio and thus the number of evaluations to perform, and the difference in runtime between an estimation and an evaluation, which is known to be very variable depending on the model and data. The more the proposal distribution is adequate and the more an evaluation is fast compared to an estimation, the greater SIR runtime gains will be.

### Comparative coverage

In the simulation examples, the coverage with SIR starting from the covariance matrix was similar to the coverage with the covariance matrix itself (Fig. 4). However, SIR outperformed the covariance matrix when starting from an inflated proposal distribution.

The default workflow did not lead to better results with SIR than with the covariance matrix in the simulation examples despite showing improvement in the real data examples. This was linked to the fact that the covariance matrix generally underestimated the true uncertainty in the simulated datasets, whereas it usually overestimated uncertainty in the real data examples. As evidenced from the investigation of the different proposal distributions, SIR is much more efficient when starting from overestimated proposal distributions than when starting from underestimated proposal distributions, which explained why SIR efficiency was reduced for simulations as compared to real data. The fact that the uncertainty based on the covariance matrix was suboptimal for some parameters, especially at low samples sizes, was not surprising. Such behaviour had been previously observed, for example with simulations with a two-compartment PK model of a dataset comprising 26 individuals and rather rich sampling where the 95 % CI did not include the true simulation value for 7 out of the 12 model parameters [36].

Suboptimal coverage could be corrected for all dataset sizes when inflations of the covariance matrix were used as proposal distributions for SIR. As expected, the optimal inflation factor needed decreased with dataset size, as the adequacy of the covariance matrix increased. Inflation factors could be easily determined based on the developed diagnostics. The same inflation factors could be used for both simulation examples, thus covariances matrices seemed to perform similarly for similar numbers of parameters (5 for the PK example and 6 for the PD example) in the investigated cases.

It is worth noting that the final SIR dOFV distribution overlaid the Chi square distribution in the simulation examples. This could indicate that the stabilization of SIR dOFV distributions below the Chi square distribution observed in the real data examples may be due to model misspecification. However, the proportion of parameters corresponding to random effects was lower in the simulation examples than in the real data examples (50–60 vs. 63–80 %). This could also explain in part why the degrees of freedom of the SIR dOFV distribution was higher in the simulations than in the real data examples, as the parameter space was less restricted.

### Performance of SIR diagnostics

The three developed diagnostics (dOFV distribution, spatial trends plot and temporal trends plot, exemplified in Fig. 2) proved highly relevant to judge whether SIR results could be considered final, both on a global (all parameters) and on a local (parameter by parameter) level. They were able to determine whether SIR settings were appropriate, i.e., whether *M/m* was sufficient to correct for the potential inadequacy of the proposal distribution. SIR results were correctly identified to be final when two conditions were fulfilled: the SIR dOFV distribution had to be at or below the Chi square distribution, with no trends apparent in the temporal trends plot. The first condition alone was not sufficient, as shown with deflations of the covariance matrix for which dOFV distributions were below the Chi square distribution but trends remained in the temporal trends plot, leading to too narrow CI. The df was a good surrogate for the stabilization of the dOFV distribution. It proved robust towards sampling noise, varying very little when performing the sampling or the resampling steps using different seeds (data not shown). More importantly, stabilization of the dOFV distribution was shown to correlate well with the actual quantities of interest, i.e., the stabilization of the parameter uncertainty distribution as summarized by RSE and CI. Marked differences in *M/m* ratios needed for stabilization were observed between parameters, in accordance to the expected appropriateness of the covariance matrix for the different parameters.

In addition to judging whether SIR settings were appropriate, the developed diagnostics informed in a qualitative manner about the adequacy of the proposal distribution: the smaller the distance between the dOFV distribution of the proposal and the Chi square distribution, the greater the adequacy of the proposal distribution. Spatial trends plots showed that symmetric proposal distributions often appeared inadequate for variances, pinpointing which parameters are typically not well described by the covariance matrix. This could in theory be used to refine the proposal distribution parameter by parameter. This was not attempted here both because performing changes at the univariate level when working with potentially correlated multivariate distributions is difficult, and because the correspondence between differences in the proportion of resamples and needed changes in the proposal distribution was not straightforward.

The evaluation of final SIR results would be much easier if the true df was known, in which case SIR results could be considered final as soon as the dOFV distribution would converge to the corresponding Chi square distribution. However, factors such as the presence of random effects, implicit or explicit parameter boundaries (e.g., variances in the positive domain or physiological boundaries) or model misspecification leave the true degrees of freedom in NLMEM models uncertain. From the experience in this work, it appears that the true degrees of freedom could potentially be derived from the convergence of the SIR dOFV distributions. The degrees of freedom stabilized around 0.8–0.9 times the total number of estimated parameters in the considered examples. To investigate whether this decrease could be linked to constraints in the parameter space, the proportion of parameter vectors simulated from the unbounded original covariance matrix that did not fulfil the constraints was computed. The rejection rate was below 10 % for the three real data examples. Whether a metric, such as the rejection rate, could be used as a correction factor to the expected degrees of freedom of the SIR resamples dOFV distribution remains to be explored. Another alternative to estimate the expected degrees of freedom could be to compute an empirical dOFV distribution obtained using stochastic simulations and re-estimations of the model. However, this is both computationally intensive and disregards the potential influence of model misspecification, which is why it was not considered here.

### Impact of *M/m* and proposal distribution on SIR results

SIR provided satisfactory results when starting from the covariance matrix with a *M/m* ratio of 5 in the real data examples. However, it was important to understand how *M/m* is impacted by the adequacy of the proposal distribution and how SIR would perform when starting from less adequate proposal distributions.

#### *M/m*

A ratio of 5 was sufficient when starting SIR from the covariance matrices of the three relatively simple PK models investigated, and could have been further reduced to two and four for two of the models. A quantitative link between *M/m* and the adequacy of the proposal distribution as measured by the df or the difference in df from the Chi square distribution, could help choosing an appropriate *M/m* to perform SIR. Differences in df between proposal distributions and Chi square distributions were around 2, 4 and 15 df under the initial SIR settings, corresponding to degrees of freedom 1.3, 1.4 and 2.4-fold higher than the Chi square degrees of freedom. It thus appeared that optimal *M/m* were around twice the ratio between the df of the proposal distribution and the df of the Chi square distribution in the investigated examples. However, this should not be regarded as an established quantitative relationship between degrees of freedom ratios and optimal *M/m* as only a very limited number of cases were investigated. The developed diagnostics thus enabled to assess whether *M/m* was sufficient a posteriori, but no quantitative relationships between the degrees of freedom of the proposal distributions and *M/m* could be established to inform the SIR procedure a priori.

#### Proposal distribution

The covariance matrix proved to be a good proposal distribution in the investigated real data examples. Starting from too narrow proposal distributions (deflations) proved problematic for SIR, as the limited number of samples in the tails of the distribution makes the expansion of parameter uncertainty very slow. Conversely, the reduction of parameter uncertainty was much easier, as evidenced by greater degrees of freedom drops between the different *M/m* for wider proposal distributions (Fig. 6). Too narrow proposal distributions could however be identified by the developed diagnostics, as they displayed dOFV distributions below the reference Chi square distribution and u-shaped trends were present in the spatial trends plots. After performing SIR with the best guess of parameter uncertainty, a change in proposal distribution should be considered instead of a change in *M/m* for increasing SIR efficiency in cases for which the proposal dOFV distribution is below the Chi square distribution and the spatial trends plot show u-shaped trends (inflate proposal distribution), or if the proposal distribution is way above the Chi square (deflate proposal distribution).

Even if the investigated proposal distributions were all derived from the covariance matrix, one major advantage of SIR is that it can be used even if the covariance matrix is not available. Any multivariate parametric distribution can indeed be used as proposal distribution. For example, one could think of using as proposal distribution the asymptotic covariance matrix obtained at an earlier stage of model development, with the uncertainty of all parameters already present at the earlier stage set to the values of the previous covariance matrix and the uncertainty of the new parameters set to an arbitrary number. Alternatively, an empirical covariance matrix obtained from a limited number of bootstrap samples (the minimum number of samples being the number of estimated parameters so that the matrix is of full rank), a generalized inverse/Cholesky matrix based on the R matrix [11] or even an educated guess (for example, 30 % RSE on all parameters and no correlations) could also be used. How to use such proposal distributions is detailed in the PsN user guide.

Modifications of the covariance matrix were used to mimic various types of proposal distributions in this work. SIR results starting from inflations greater than or equal to 1 had not converged at the highest tested *M/m* ratio of 10, which indicated that higher ratios would be needed to achieve final SIR results if the proposal distribution is heavily misspecified (Fig. 6). To reach final results efficiently, modifications of the proposal distribution looked like a better alternative than increasing *M/m*: changes in degrees of freedom were constant and high between proposal distributions (increases of 10, 12 and 28 df per inflation unit of 1 for pefloxacin, phenobarbital and moxonidine respectively) whereas changes in degrees of freedom between *M/m* ratios were low and decreased drastically with increasing *M/m* and decreasing inflation factor (highest changes of 7, 3 and 2 df for twofold inflated proposal distributions).

Another dimension of the proposal distribution, which deserves attention but was not investigated in this work, is the correlation between parameter uncertainty distributions. Just as it is difficult for SIR to compensate for too narrow proposal distributions, reducing the absolute value of correlations is much more difficult than increasing it, as the number of vectors leading to low correlations will be low when simulated under high correlations. Correlations were low in all investigated real data examples and were kept unchanged under inflations/deflations of the proposal distribution. However, high correlations are often observed, for example between maximum effect (Emax) and concentration leading to half the maximum effect (EC50) in Emax models. Such correlations may heavily restrict both the size and the shape of the investigated parameter space. Causes and consequences of potentially misspecified correlations are unclear and few examples investigating this have been published in NLMEM [37]. Reducing correlations between highly correlated parameter uncertainties (while increasing the number of initial samples) should nevertheless be considered when performing SIR to guard against too confining constraints. More generally, the covariance matrix is known to be a bad approximation of the true uncertainty in the presence of high curvature (i.e., non-normal uncertainty, which happens for example when sample sizes are low, or when parameters are very nonlinear) or collinearity between parameters, especially if the correlation is nonlinear (such as for parameters of the sigmoid Emax model) [38]. It is thus highly advised to use parameterizations that minimize such issues, as proposed in Reeve et al. [8].

At this point it is relevant to mention that the ultimate diagnostic to test whether SIR results are final would be to perform SIR using the final SIR results as proposal distribution and see no difference between this proposal distribution and the new SIR results. This thought, i.e. running iterative SIRs until convergence is reached, is currently under investigation and is readily available in PsN 4.6.0 (released May 2016).

## Conclusion

The SIR method was applied to parameter uncertainty estimation in NLMEM and diagnostics to assess the appropriateness of SIR settings were developed. SIR is fast, does not require parameter estimation or distributional assumptions, and is thus expected to perform better than previously available methods for parameter uncertainty assessment in many cases, including small datasets, highly nonlinear models or meta-analysis. An implementation of the SIR method is readily available in the PsN software package. Further improvements to the SIR workflow, including iterative SIR procedures and more flexible proposal distributions, are under development.

## Electronic supplementary material

Below is the link to the electronic supplementary material.

Supplementary material 1 (DOCX 26 kb)

Supplementary material 2 (DOCX 58 kb)

Supplementary material 3 (DOCX 22 kb)

Supplementary material 4 (DOCX 323 kb)

Supplementary material 5 (DOCX 357 kb)

Supplementary material 6 (DOCX 61 kb)

## References

[1] Lalonde RL, Kowalski KG, Hutmacher MM, Ewy W, Nichols DJ, Milligan PA, Corrigan BW, Lockwood PA, Marshall SA, Benincosa LJ, Tensfeldt TG, Parivar K, Amantea M, Glue P, Koide H, Miller R (2007). Model-based drug development. Clin Pharmacol Ther.

[2] Jonsson EN, Sheiner LB (2002). More efficient clinical trials through use of scientific model-based statistical tests. Clin Pharmacol Ther.

[3] Kraiczi H, Frisen M (2005). Effect of uncertainty about population parameters on pharmacodynamics-based prediction of clinical trial power. Contemp Clini Trials.

[4] Santen G, Danhof M, Pasqua OD (2008) Uncertainty and decision making in clinical development: the impact of an interim analysis based on the posterior predictive power on depression trials. http://tucson2008.go-acop.org/pdfs/DellaPasqua2.pdf. Accessed 16 Mar 2015

[5] Waterhouse T, Benson C, Chiang A, Tang C (2009) Using uncertainty in exposure-response modeling and simulation to select phase II doses. http://2009.go-acop.org/sites/all/assets/webform/Waterhouse%20ACOP%202009%20poster.pdf. Accessed 2015 Mar 16

[6] Gupta M, Wade K, Jayaraman B, Barrett J (2006) Optimizing fluconazole dosing in preterm neonates based on simulations from posterior parameter (uncertainty) distributions. PAGE Abstracts of the Annual Meeting of the Population Approach Group in Europe

[7] Davidian MGD (1995). Nonlinear models for repeated measurement data.

[8] Reeve R, Turner JR (2013). Pharmacodynamic models: parameterizing the Hill Equation, Michaelis-Menten, the logistic curve, and relationships among these models. J Biopharm Stat.

[9] Bates DM, Watts DG (1980). Relative curvature measures of nonlinearity. J Royal Stat Soc.

[10] Cook RD, Witmer JA (1985). A note on parameter-effects curvature. J Am Stat Assoc.

[11] Gill J, King G, Altman MJG, McDonald MP (2004). Numerical issues involved in inverting hessian matrices. Numerical issues in statistical computing for the social scientist.

[12] Efron B (1979). Bootstrap methods: another look at the jackknife. Ann Stat.

[13] Thai HT, Mentre F, Holford NH, Veyrat-Follet C, Comets E (2014). Evaluation of bootstrap methods for estimating uncertainty of parameters in nonlinear mixed-effects models: a simulation study in population pharmacokinetics. J Pharmacokinet Pharmacodyn.

[14] Leary R (2013) A fast bootstrap method using EM posteriors. In: PAGE 22, Glasgow

[15] Niebecker R, Karlsson M (2013) Are datasets for nonlinear mixed-effects models large enough for a bootstrap to provide reliable parameter uncertainty distributions? In: PAGE 22 Meeting. University of Strathclyde, Glasgow

[16] Sheiner LB (1986). Analysis of pharmacokinetic data using parametric models. III. Hypothesis tests and confidence intervals. J Pharmacokinet Biopharm.

[17] Lindbom L, Pihlgren P, Jonsson EN (2005). PsN-Toolkit–a collection of computer intensive statistical methods for non-linear mixed effect modeling using NONMEM. Comput Methods Progr Biomed.

[18] Denney W (2012) N-dimensional likelihood profiling: an efficient alternative to bootstrap. In: PAGE 21 Meeting, Venice

[19] Rubin D, Bernardo J, DeGroot M, Lindley D, Smith A (1988). Using the SIR algorithm to simulate posterior distributions. Bayesian statistics 3.

[20] Gelfand AE, Smith AFM (1990). Sampling-based approaches to calculating marginal densities. J Am Stat Assoc.

[21] Thomas N, Gan N (1997). Generating multiple imputations for matrix sampling data analyzed with item response models. J Educ Behav Stat.

[22] Smith AFM, Gelfand AE (1992). Bayesian statistics without tears: a sampling-resampling perspective. Am Stat.

[23] Poole D, Raftery AE (2000). Inference for deterministic simulation models: the bayesian melding approach. J Am Stat Assoc.

[24] Raftery AE, Bao L (2010). Estimating and projecting trends in HIV/AIDS generalized epidemics using incremental mixture importance sampling. Biometrics.

[25] Heitjan DF, Li H (2004). Bayesian estimation of cost-effectiveness: an importance-sampling approach. Health Econ.

[26] Vanlier J, Tiemann CA, Hilbers PA, van Riel NA (2012). A Bayesian approach to targeted experiment design. Bioinformatics.

[27] Karlsson MO, Jonsson EN, Wiltse CG, Wade JR (1998). Assumption testing in population pharmacokinetic models: illustrated with an analysis of moxonidine data from congestive heart failure patients. J Pharmacokinet Biopharm.

[28] Wahlby U, Thomson AH, Milligan PA, Karlsson MO (2004). Models for time-varying covariates in population pharmacokinetic-pharmacodynamic analysis. Br J Clin Pharmacol.

[29] Grasela TH, Donn SM (1985). Neonatal population pharmacokinetics of phenobarbital derived from routine clinical data. Dev Pharmacol Ther.

[30] Wilks S (1937). The large-sample distribution of the likelihood ratio for testing composite hypotheses. Am Math Soc.

[31] Wahlby U, Jonsson EN, Karlsson MO (2001). Assessment of actual significance levels for covariate effects in NONMEM. J Pharmacokinet Pharmacodyn.

[32] Beal S, Sheiner LB, Boeckmann A, Bauer RJ (2009). NONMEM user’s guides (1989–2009).

[33] R Core Team (2015). R: A language and environment for statistical computing.

[34] Dartois C, Laveille C, Trnachand B, Tod M, Girard P (2004) What is the value of uncertainty parameter estimates provided by different population PK methods? PAGE Abstracts of the Annual Meeting of the Population Approach Group in Europe PAGE 13

[35] Donaldson JR, Schnabel RB (1987). Computational experience with confidence regions and confidence intervals for nonlinear least squares. Technometrics.

[36] Yafune A, Ishiguro M (1999). Bootstrap approach for constructing confidence intervals for population pharmacokinetic parameters. I: A use of bootstrap standard error. Stat Med.

[37] Garnett C, Holford N (2004). The relative importance of between-subject correlation of population parameters compared with estimation correlation when applied to pharmacokinetic simulation. Clin Pharmacol Ther.

[38] Meyers R (1990). Classical and modern regression with applications.

