# Supplementary material for: Improving the estimation of parameter uncertainty distributions in nonlinear mixed effects models using sampling importance resampling
Source: J Pharmacokinet Pharmacodyn. 2016 Oct 11;43(6):583–96. doi: 10.1007/s10928-016-9487-8 (PMC5110709; doi:10.1007/s10928-016-9487-8)
Supplement: Supplementary file 1 — Supplementary material 1 (DOCX 26 kb) [file 10928_2016_9487_MOESM1_ESM.docx]

**Online Resource 1: Details of the SIR procedure**

| Article title | Improving the Estimation of Parameter Uncertainty Distributions in Nonlinear Mixed Effects Models using Sampling Importance Resampling |
| --- | --- |
| Journal name | Journal of Pharmacokinetics and Pharmacodynamics |
| Author names | Anne-Gaëlle Dosne^1^, Martin Bergstrand^1^, Kajsa Harling^1^, Mats O Karlsson^1^ |
| Author affiliations | ^1^Department of Pharmaceutical Biosciences, Uppsala University, P.O. Box 591, 751 24 Uppsala, Sweden |
| Corresponding author | Anne-Gaëlle Dosne: [annegaelle.dosne@farmbio.uu.se](mailto:annegaelle.dosne@farmbio.uu.se) |

With SIR, the likelihood of each resampled vector *θ_i_* should be equal to *p_true_(θ_i_*). However, since *p_true_(θ)* is unknown, *p_true_(θ_i_*) cannot be directly evaluated. However, we can use a surrogate of *p_true_(θ)*, called the proposal uncertainty distribution *h(θ)*, to evaluate a likelihood which is proportional to *p_true_(θ)*.

1. **Step 1 (sampling)**: The probability of sampling each vector, *p_sampling_(θ)*, is proportional to *h(θ).*
2. **Step 2 (importance weighting)**: The importance ratio *IR_i_* defines the probability of the vector being resampled in the next step as *p_resampling_(θ_i_)= IR_i_ / ΣIR_._* The resampling probability *p_resampling_(θ_i_ | θ_i_ is sampled)* is proportional to *p_true_(θ_i_)/h(θ_i_)* and therefore *IR_i_* = *p'(θ_i_)/h'(θ_i_),* with *p'(θ_i_)* and *h'(θ_i_)* respectively proportional to *p_true_(θ_i_)* and *h(θ_i_).* Any multiple of *p_true_(θ_i_)* and *h(θ_i_)* in the *IR* will give the same normalized resampling probability since the sum of resampling probabilities is 1. However, the resampling probability will be proportional to *IR* only as *M/m* tends towards infinity. For example, in the extreme case of *M=m*, *p_resampling_(θ_i_ | θ_i_ is sampled)* =1, i.e. all samples are resampled. In the context of NLMEM, *p’(θ_i_)* was set to the likelihood of the observed data given the parameter vector *θ_i_*, which was expressed relative to the likelihood of the data given the vector of maximum likelihood estimates *θ_ML_*, as displayed in Equation A1. Likelihoods were calculated based on the differences in OFV (dOFV) obtained in NONMEM with the FOCEI method.

| $p'\left( \theta_{i} \right)=\frac{exp\left( \frac{-1}{2}{OFV}_{i} \right)}{exp\left( \frac{-1}{2}{OFV}_{ML} \right)}=exp\left( \frac{-1}{2}{dOFV}_{i} \right)$ | Equation A1 |
| --- | --- |
| *where* $p'\left( \theta_{i} \right)$ *is the relative likelihood of the data given the parameter vector* $\theta_{i}$*,* ${OFV}_{i}$ *is the objective function value for* $\theta_{i}$*,* ${OFV}_{ML}$ *is the objective function value for* $\theta_{ML}$ *and* ${dOFV}_{i}$*is the difference between* ${OFV}_{i}$*and* ${OFV}_{ML}.$ | |

The denominator *h’(θ_i_)* of *IR_i_* was computed as the likelihood of the parameter vector *θ_i_* in the proposal uncertainty distribution *h(θ),* which was expressed relative to that of *θ_ML_* in the same distribution. These likelihoods were based on the formula for the probability density function (PDF) of a multivariate normal distribution (Equation A2 and A3).

| ${PDF}_{i}=\frac{1}{{2\pi}^{p/2}{det\left( h\left( \theta\right) \right)}^{1/2}}exp\left( \frac{-1}{2}\left( \theta_{i}-\theta_{ML} \right){h\left( \theta\right)}^{-1}\left( \theta_{i}-\theta_{ML} \right)^{T} \right)$ | Equation A2 |
| --- | --- |
| $h'\left( \theta_{i} \right)={relPDF}_{i}=\frac{{PDF}_{i}}{{PDF}_{ML}}=exp\left( \frac{-1}{2}\left( \theta_{i}-\theta_{ML} \right){h\left( \theta\right)}^{-1}\left( \theta_{i}-\theta_{ML} \right)^{T} \right)$ | Equation A3 |
| *Where* ${PDF}_{i}$ *is the likelihood of the parameter vector* $\theta_{i}$ *given the multivariate normal distribution* $h\left( \theta\right)$*, p is the length of the* $\theta_{i}$ *vector,* $det(h\left( \theta\right))$ *is the determinant of* $h\left( \theta\right)$*,* $\theta_{ML}$ *is the vector of maximum likelihood estimates for the model and data at hand,* ${h\left( \theta\right)}^{-1}$*is the inverse of* $h\left( \theta\right)$*, T stands for vector transpose,* $h^{'}\left( i \right)$ *is the denominator of IR_i_ and* ${relPDF}_{i}$ *is the likelihood of the parameter vector* $\theta_{i}$ *given* $h\left( \theta\right)$ *relative to the likelihood of* $\theta_{ML}$ *given* $h\left( \theta\right).$ | |

The resulting formula for the calculation of *IR_i_* is stated in Equation A4.

| ${IR}_{i}=\frac{p'\left( \theta_{i} \right)}{h'\left( \theta_{i} \right)}=\frac{exp\left( \frac{-1}{2}{dOFV}_{i} \right)}{{relPDF}_{i}}$ | Equation A4 |
| --- | --- |

If two samples *θ_i_* and *θ_j_* have equal sampling probability, *h’(θ_i_)=h’(θ_j_)*, but different likelihoods given the data, *p’(θ_i_)>p’(θ_j_)*, then the sample with higher likelihood will have greater IR, *IR_i_*>*IR_j._*. If for two samples *θ_k_* and *θ_l_* have equal likelihoods given the data, *p’(θ_k_)=p’(θ_l_)*, but different sampling probabilities *h’(θ_k_)<h’(θ_l_)*, the sample with lower sampling probability will have greater IR, *IR_k_*>*IR_l._*. If the proposal *h(θ)* is proportional to the likelihood and hence the true uncertainty, *IR* will be constant and all samples will be resampled with equal probability.

1. **Step 3 (resampling)**: in the last step, *m* parameter vectors were resampled from the pool of *M* simulated vectors with probabilities proportional to their *IR*.
