# Supplementary material for: Improving the estimation of parameter uncertainty distributions in nonlinear mixed effects models using sampling importance resampling
Source: J Pharmacokinet Pharmacodyn. 2016 Oct 11;43(6):583–96. doi: 10.1007/s10928-016-9487-8 (PMC5110709; doi:10.1007/s10928-016-9487-8)
Supplement: Supplementary file 2 — Supplementary material 2 (DOCX 58 kb) [file 10928_2016_9487_MOESM2_ESM.docx]

Online Resource 2: Investigation of replacement as an additional SIR setting

| Article title | Improving the Estimation of Parameter Uncertainty Distributions in Nonlinear Mixed Effects Models using Sampling Importance Resampling |
| --- | --- |
| Journal name | Journal of Pharmacokinetics and Pharmacodynamics |
| Author names | Anne-Gaëlle Dosne^1^, Martin Bergstrand^1^, Kajsa Harling^1^, Mats O Karlsson^1^ |
| Author affiliations | ^1^Department of Pharmaceutical Biosciences, Uppsala University, P.O. Box 591, 751 24 Uppsala, Sweden |
| Corresponding author | Anne-Gaëlle Dosne: [annegaelle.dosne@farmbio.uu.se](mailto:annegaelle.dosne@farmbio.uu.se) |

Performing SIR with replacement at the resampling step was investigated as a potential way of making SIR more efficient. Three replacement strategies were investigated: no replacement, limited replacement (with a cap at 5, i.e. each parameter vector was allowed to be resampled maximum of 5 times) and unlimited replacement. The degrees of freedom estimated when using limited and unlimited replacement during the resampling step of the SIR procedure are displayed in the middle and right panels of Fig. A1.


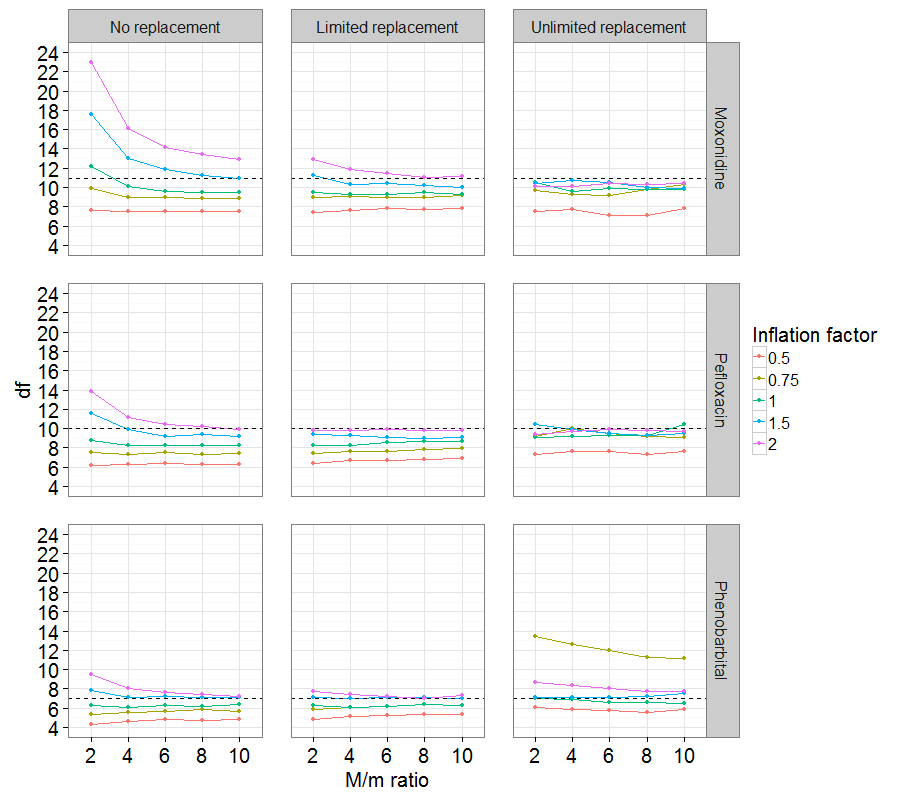


**Fig. A1** SIR convergence over investigated settings: degrees of freedom (df) versus number of initial samples *M* by inflation factor and replacement alternative for the three real data examples

Final dOFV curves using limited resampling had lower degrees of freedom than those obtained without replacement at identical *M* and inflation factor. Limited resampling led to faster dOFV stabilization, with optimal *M/m* ratios for the uninflated covariance matrix at 2 for all models instead of 6, 4 and 2 previously. Starting from different proposals with limited replacement led to more similar SIR results at *M*/*m* = 10 than without replacement (df ranges of 3, 3 and 2 versus 5, 4 and 2), with some proposals converging. Final degrees of freedom under the original covariance matrix with limited replacement was close to that without replacement, indicating that SIR results were consistent between these strategies. Using unlimited replacement showed a more erratic behaviour. For moxonidine and pefloxacin, all starting proposals except the 0.5-deflation were within 2 df of each other from the lowest *M/m* ratio on: results between proposals were closer than with the other replacement strategies. However, within a single proposal, degrees of freedom showed high variation between *M/m,* which made the assessment of the optimal *M/m* difficult. For phenobarbital, unlimited replacement did not lead to satisfactory SIR results: for all proposals, the degrees of freedom were higher than with the previous resampling strategies, and the spread between the proposals was greater than before. The impact of replacement on parameter RSE and CI bounds in the moxonidine example when *M/m* = 10 and no inflation is displayed in Fig. A2.


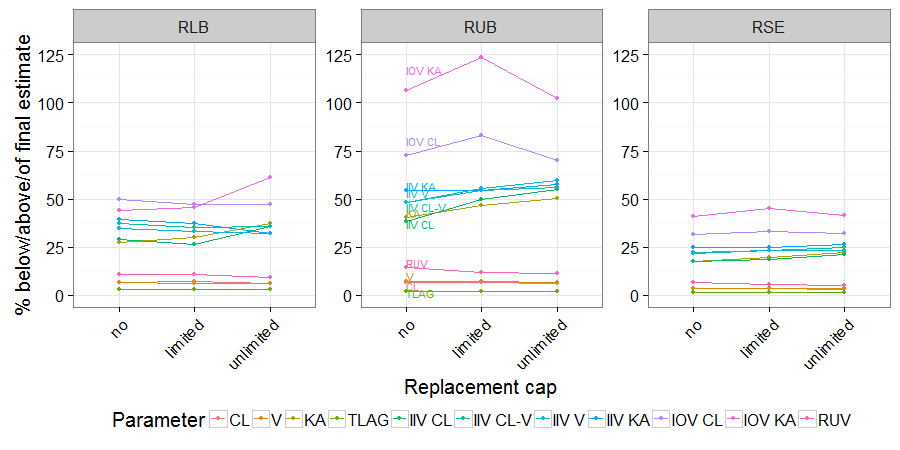


**Fig. A2** 95% CI bounds and RSE of the moxonidine model parameters with the different replacement strategies. RLB: relative lower bound, RUB: relative upper bound, RSE: relative standard error. SIR performed with 10,000 initial samples and without inflation. Y-axis interpretation: After performing SIR with no replacement, the lower bound of the 95% CI of IOV KA (pink) is 44% below the final estimate, the upper bound is 106% above the final estimate and the corresponding RSE is 41%.

Allowing replacement at the resampling step led to faster stabilization of the degrees of freedom, but could lead to greater variability and erratic shapes when replacement was unlimited. The percentage of unique models when allowing unlimited replacement was below 50% for moxonidine and pefloxacin, with between 6 and 15% of the resampled vectors corresponding to a single model when using 10,000 initial samples and no inflation. This was thought to be potentially prone to bias in the resulting uncertainty estimate. The percentage was higher (between 60% and 85%) when replacement was capped at 5, but this was still considered at risk of bias. In conclusion, limited replacement could increase SIR efficiency but due to the lack of guidance regarding the appropriate cap and its consequence on potential bias, it was not advised to use replacement during the SIR resampling step.
