# Supplementary material for: Improving the estimation of parameter uncertainty distributions in nonlinear mixed effects models using sampling importance resampling
Source: J Pharmacokinet Pharmacodyn. 2016 Oct 11;43(6):583–96. doi: 10.1007/s10928-016-9487-8 (PMC5110709; doi:10.1007/s10928-016-9487-8)
Supplement: Supplementary file 3 — Supplementary material 3 (DOCX 22 kb) [file 10928_2016_9487_MOESM3_ESM.docx]

Online Resource 3 Details of the models and designs

| Article title | Improving the Estimation of Parameter Uncertainty Distributions in Nonlinear Mixed Effects Models using Sampling Importance Resampling |
| --- | --- |
| Journal name | Journal of Pharmacokinetics and Pharmacodynamics |
| Author names | Anne-Gaëlle Dosne^1^, Martin Bergstrand^1^, Kajsa Harling^1^, Mats O Karlsson^1^ |
| Author affiliations | ^1^Department of Pharmaceutical Biosciences, Uppsala University, P.O. Box 591, 751 24 Uppsala, Sweden |
| Corresponding author | Anne-Gaëlle Dosne: [annegaelle.dosne@farmbio.uu.se](mailto:annegaelle.dosne@farmbio.uu.se) |

| **Simulation example 1: PK 1CMT** | | | **Simulation example 2: PD Emax** | | |
| --- | --- | --- | --- | --- | --- |
| **Model** | | | | | |
| CL (first order elimination) | 1 | | E0 | | 10 |
| V | 1 | | EMAX | | 100 |
| IIV CL | 30% | | ED50 | | 5 |
| IIV V | 30% | | IIV E0 | | 30% |
| RUV, additive on log scale | 20% | | IIV ED50 | | 30% |
|  |  |  | RUV, proportional | | 10% |
| **Study design** | | | | | |
| Single dose of 100 mass units  Sampling after 0.25, 0.5, 1 and 2 time units. | | | Multiple doses of 0, 2.5, 5, 15 and 2 dose units. | | |
| ID – obs/ID: 20 - 4 | | | ID – obs/ID: 20 - 4 | | |
| ID – obs/ID: 50 - 4 | | | ID – obs/ID: 50 - 4 | | |
| ID – obs/ID: 200 - 4 | | | ID – obs/ID: 200 – 4 | | |
| **Real data example 1:**  **Moxonidine** | | **Real data example 2:**  **Pefloxacin** | | **Real data example 3:**  **Phenobarbital** | |
| **Model** | | | | | |
| oral PK 1-CMT with lag-time first order absorption and elimination  11 parameters:  CL, V, KA, TLAG,  IIV CL, IIV V, IIV CL-V, IIV KA,  IOV CL, IOV KA,  additive RUV on log scale | | i.v. PK 1-CMT first order elimination  10 parameters:  CL, V,  IIV CL, IIV V, IIV CL-V,  covariate effect on CL (CLCR)  IOV CL, IOV V, IOV CL-V  proportional RUV | | i.v. PK 1-CMT first order elimination  7 parameters:  CL, V,  IIV CL, IIV V  weight effects on CL and V (EXP)  additive RUV | |
| **Study design** | | | | | |
| multiple doses  2 occasions | | multiple doses  3 occasions | | multiple doses  1 occasion | |
| ID – Nobs (obs/ID): 74 – 1021 (14) | | ID – Nobs (obs/ID): 74 – 337 (5) | | ID – Nobs (obs/ID): 59 – 155 (3) | |
| CL: clearance, V: volume of distribution, TLAG: lag-time, KA: absorption rate, IIV: inter-individual variability (exponential, expressed as coefficient of variation in %), IOV: inter-occasion variability (exponential, expressed as coefficient of variation in %), RUV: residual unexplained variability (expressed as coefficient of variation in %), ID: individuals, obs/ID: number of observations per individual, Nobs: total number of observations, CMT: compartment, i.v.:intravenous. | | | | | |

**NONMEM control file for the moxonidine example**

$PROBLEM MOXONIDINE PK, FINAL ESTIMATES, ALL DATA

$INPUT ID VISI XAT2=DROP DGRP=DROP DOSE=DROP FLAG=DROP ONO=DROP

XIME=DROP DVO=DROP NEUY SCR AGE SEX NYHA WT

DROP ACE DIG DIU NUMB=DROP TAD TIME VIDD=DROP CRCL AMT SS

II DROP CMT=DROP CONO=DROP DV EVID=DROP OVID=DROP

DROP SHR2=DROP NYHDI=DROP

$DATA mx20.csv IGNORE=#

$SUBROUTINE ADVAN2 TRANS1

$PK

;-----------OCCASIONS----------

VIS3 = 0

IF(VISI.EQ.3) VIS3 = 1

VIS8 = 0

IF(VISI.EQ.8) VIS8 = 1

;----------IOV--------------------

KPCL = VIS3*ETA(4)+VIS8*ETA(5)

KPKA = VIS3*ETA(6)+VIS8*ETA(7)

;---------- PK model ------------------

TVCL = THETA(1)

TVV = THETA(2)

CL = TVCL*EXP(ETA(1)+KPCL)

V = TVV*EXP(ETA(2))

KA = THETA(3)*EXP(ETA(3)+KPKA)

ALAG1 = THETA(4)

K = CL/V

S2 = V

$ERROR

IPRED = LOG(.025)

IF(F.GT.0) IPRED = LOG(F)

W = SQRT(SIGMA(1,1))

IRES = IPRED-DV

IWRES = IRES/W

Y = IPRED+EPS(1)

$THETA (0,26.6815) ; CL

$THETA (0,110.275) ; V

$THETA (0,4.49465) ; KA

$THETA (0,0.240122,0.25) ; TLAG

$OMEGA BLOCK(2) 0.0444 ; IIV CL

0.027 ; COV CL-V

0.0241 ; IIV V

$OMEGA BLOCK(1) 3.0 ; IIV KA

$OMEGA BLOCK(1) 0.0165 ; IOV CL

$OMEGA BLOCK(1) SAME ; IOV CL

$OMEGA BLOCK(1) 0.495 ; IOV KA

$OMEGA BLOCK(1) SAME ; IOV KA

$SIGMA 0.1

$ESTIMATION METHOD=1 MAXEVALS=9999

$COV

NONMEM control file for the pefloxacin example

$PROBLEM pefloxacin data intraindividual modeling

$INPUT ID OCC DV TIME AMT RATE ADDL II WT AGE CLCR SEX CEN BIL SBP

$DATA pefdata.csv IGN=@

$SUBROUTINE ADVAN=ADVAN1

$PK

OCC1=0

OCC2=0

OCC3=0

IF (OCC.EQ.0) OCC1=1

IF (OCC.EQ.1) OCC2=1

IF (OCC.EQ.2) OCC3=1

IF(NEWIND.NE.2) BCLCR=CLCR

IF(NEWIND.NE.2) BWT =WT

DCLCR=CLCR-BCLCR

DWT =WT-BWT

TVCL=THETA(1)*(1+THETA(4)*(CLCR-100))

CL=TVCL*EXP(ETA(1)+OCC1*ETA(3)+OCC2*ETA(5)+OCC3*ETA(7))

TVV=THETA(2)*(WT/70)

V =TVV *EXP(ETA(2)+OCC1*ETA(4)+OCC2*ETA(6)+OCC3*ETA(8))

S1=V

K=CL/V

$ERROR

IPRED=F

W =THETA(3)*IPRED

IWRES=(DV-IPRED)/W

Y=IPRED + W*ERR(1)

$THETA (0,3.42508) ; 1 TVCL

$THETA (0,66.1332) ; 2 TVV

$THETA (0,0.142616) ; 3 RV

$THETA (0,0.0041804,.0099) ; 4 CRCL on CL

$OMEGA BLOCK(2)

0.101439

0.0561715 0.054985

$OMEGA BLOCK(2)

0.136314

-0.0122524 0.00811249

$OMEGA BLOCK(2) SAME

$OMEGA BLOCK(2) SAME

$SIGMA 1 FIX

$ESTIMATION SIG=3 MAXEVAL=3000 PRINT=10 MSFO=msf1 METHOD=1 INTER

$COVARIANCE

NONMEM **control** file for the phenobarbital example

$PROBLEM PHENOBARB additive model

;$ABBREVIATED COMRES=2

$INPUT ID TIME AMT WT APGR DV

$DATA PHENO.dta IGNORE=@

$SUBROUTINE ADVAN1 TRANS2

$PK

TVCL = THETA(1)*(WT/3)**THETA(4) ; typical value of CL

TVV = THETA(2)*(WT/3)**THETA(5)

CL = TVCL*EXP(ETA(1)) ; individual value of CL

V = TVV*EXP(ETA(2)) ; individual value of V

S1 = V

$ERROR

IPRED = F ; individual predicion

IRES = DV - F ; individual residual

W = THETA(3) ; additive residual error

IF(W.EQ.0) W = 1

IWRES = IRES/W ; individual weighed residual

;IF(ICALL.EQ.4) THEN

; Y=DV

;ELSE

Y = IPRED+ERR(1)*W

;ENDIF

$THETA (0,.005)

$THETA (0,1.45)

$THETA (0, 5)

$THETA (0, .75)

$THETA (0, 1)

$OMEGA 0.228 ; variance for ETA(1), initial estimate

$OMEGA 0.146 ; variance for ETA(2), initial estimate

$SIGMA 1 FIX ; initial estimate

$ESTIMATION METHOD=1 MAXEVAL=9999 ; FOCE calculation method

$COV
