# Supplementary material for: Improving the estimation of parameter uncertainty distributions in nonlinear mixed effects models using sampling importance resampling
Source: J Pharmacokinet Pharmacodyn. 2016 Oct 11;43(6):583–96. doi: 10.1007/s10928-016-9487-8 (PMC5110709; doi:10.1007/s10928-016-9487-8)
Supplement: Supplementary file 4 — Supplementary material 4 (DOCX 323 kb) [file 10928_2016_9487_MOESM4_ESM.docx]

**Online Resource 4: Diagnostic plots for the pefloxacin and phenobarbital models**

| Article title | Improving the Estimation of Parameter Uncertainty Distributions in Nonlinear Mixed Effects Models using Sampling Importance Resampling |
| --- | --- |
| Journal name | Journal of Pharmacokinetics and Pharmacodynamics |
| Author names | Anne-Gaëlle Dosne^1^, Martin Bergstrand^1^, Kajsa Harling^1^, Mats O Karlsson^1^ |
| Author affiliations | ^1^Department of Pharmaceutical Biosciences, Uppsala University, P.O. Box 591, 751 24 Uppsala, Sweden |
| Corresponding author | Anne-Gaëlle Dosne: [annegaelle.dosne@farmbio.uu.se](mailto:annegaelle.dosne@farmbio.uu.se) |
|  |  |

**(a) Pefloxacin**


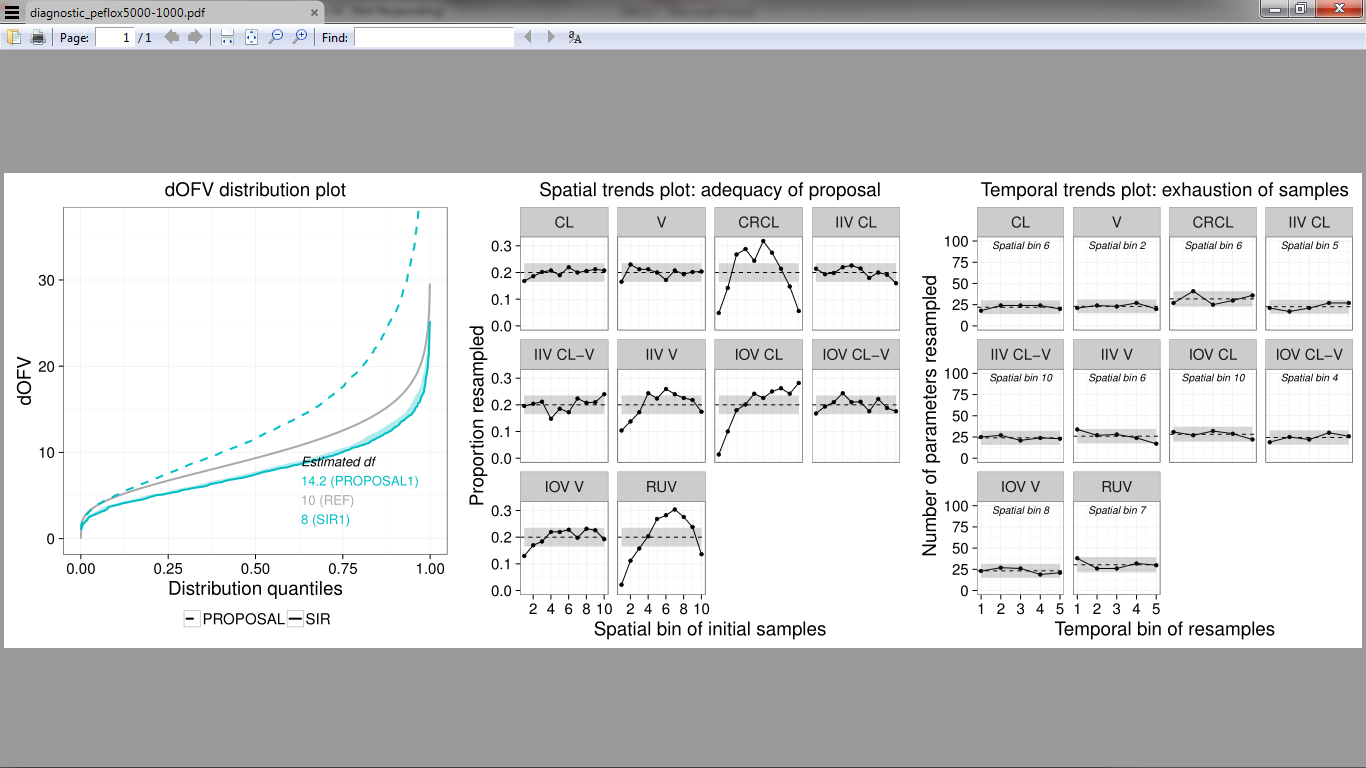


**(b) Phenobarbital**


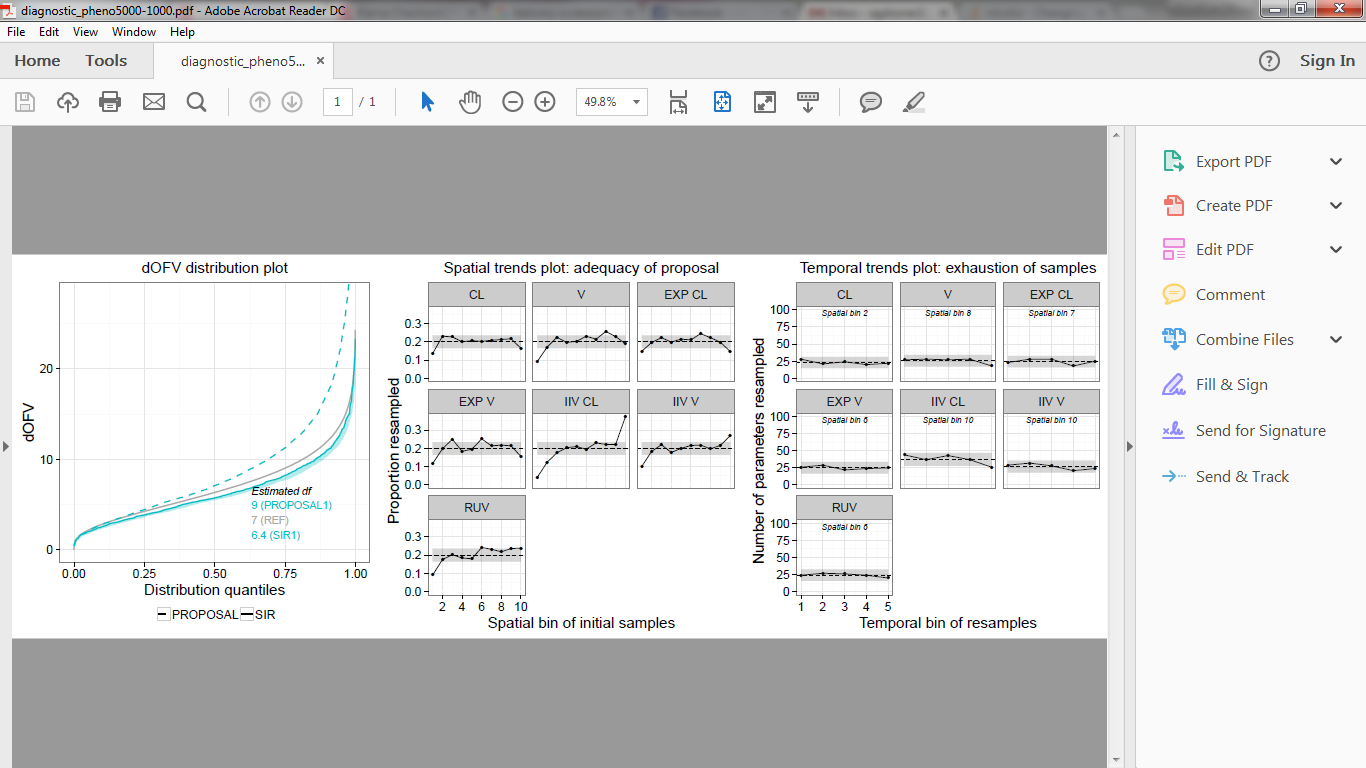


**Fig. A3** Diagnostic plots for the SIR results of the pefloxacin (panel a) and phenobarbital (panel b) real data examples: dOFV distribution plot (left panel), spatial trends plot (middle panel) and temporal trends plot (right panel).

The dOFV plot displays three dOFV distributions: the proposal distribution, i.e. the covariance matrix (blue dotted line), the reference chi-square distribution (with degrees of freedom equal to the number of estimated parameters, grey full line), and the SIR distribution (blue full line). It informs about the qadequacy of the proposal and whether SIR results can be considered final. The value of the dOFV (y-axis) is plotted for each percentile of the distribution (x-axis). The blue shaded area represents the resampling noise around the SIR distribution (as 95% CI). The resampling noise was calculated by performing the resampling step 2000 times. The estimated degrees of freedom for each distribution are displayed in the bottom right corner.

The spatial trends plot displays the proportion of parameters resampled in each spatial bin and informs about the adequacy of the proposal. Stochastic noise was approximated by the 95% CI of a normal distribution with the estimated proportion *p* as mean and √p*(1-*p*)/*N* as standard error*.*

The temporal trends plot displays the number of parameters resampled in each time bin and informs about the exhaustion of samples. Stochastic noise was approximated by the 95% confidence interval of a normal distribution with the estimated proportion *p* as mean and √p*(1-*p*)/*N* as standard error, the proportion being then back-transformed to the number of resampled parameters given the available number of parameters.
