# Supplementary material for: Improving the estimation of parameter uncertainty distributions in nonlinear mixed effects models using sampling importance resampling
Source: J Pharmacokinet Pharmacodyn. 2016 Oct 11;43(6):583–96. doi: 10.1007/s10928-016-9487-8 (PMC5110709; doi:10.1007/s10928-016-9487-8)
Supplement: Supplementary file 5 — Supplementary material 5 (DOCX 357 kb) [file 10928_2016_9487_MOESM5_ESM.docx]

**Online Resource 5: Comparative 95% confidence intervals of the real data examples pefloxacin and phenobarbital**

| Article title | Improving the Estimation of Parameter Uncertainty Distributions in Nonlinear Mixed Effects Models using Sampling Importance Resampling |
| --- | --- |
| Journal name | Journal of Pharmacokinetics and Pharmacodynamics |
| Author names | Anne-Gaëlle Dosne^1^, Martin Bergstrand^1^, Kajsa Harling^1^, Mats O Karlsson^1^ |
| Author affiliations | ^1^Department of Pharmaceutical Biosciences, Uppsala University, P.O. Box 591, 751 24 Uppsala, Sweden |
| Corresponding author | Anne-Gaëlle Dosne: [annegaelle.dosne@farmbio.uu.se](mailto:annegaelle.dosne@farmbio.uu.se) |

| 1. **Pefloxacin**   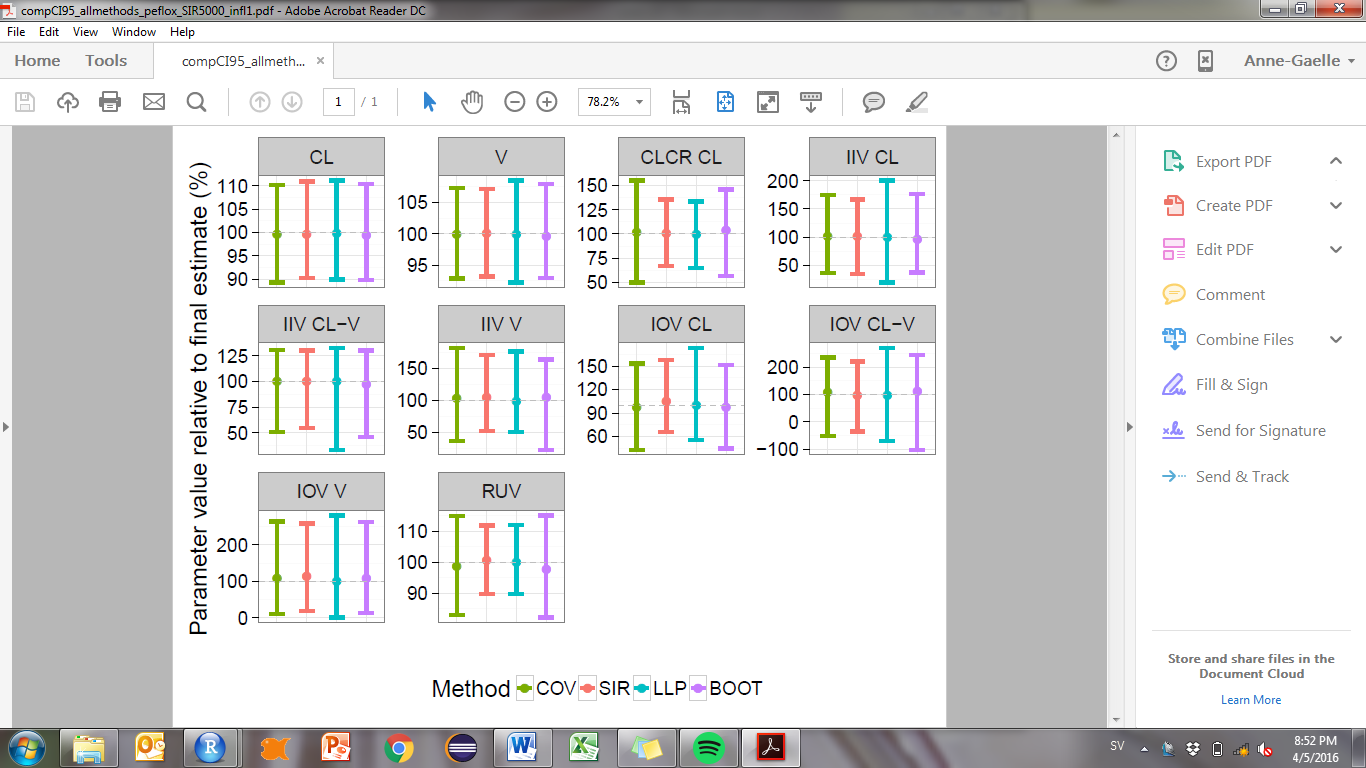 | 1. **Phenobarbital**   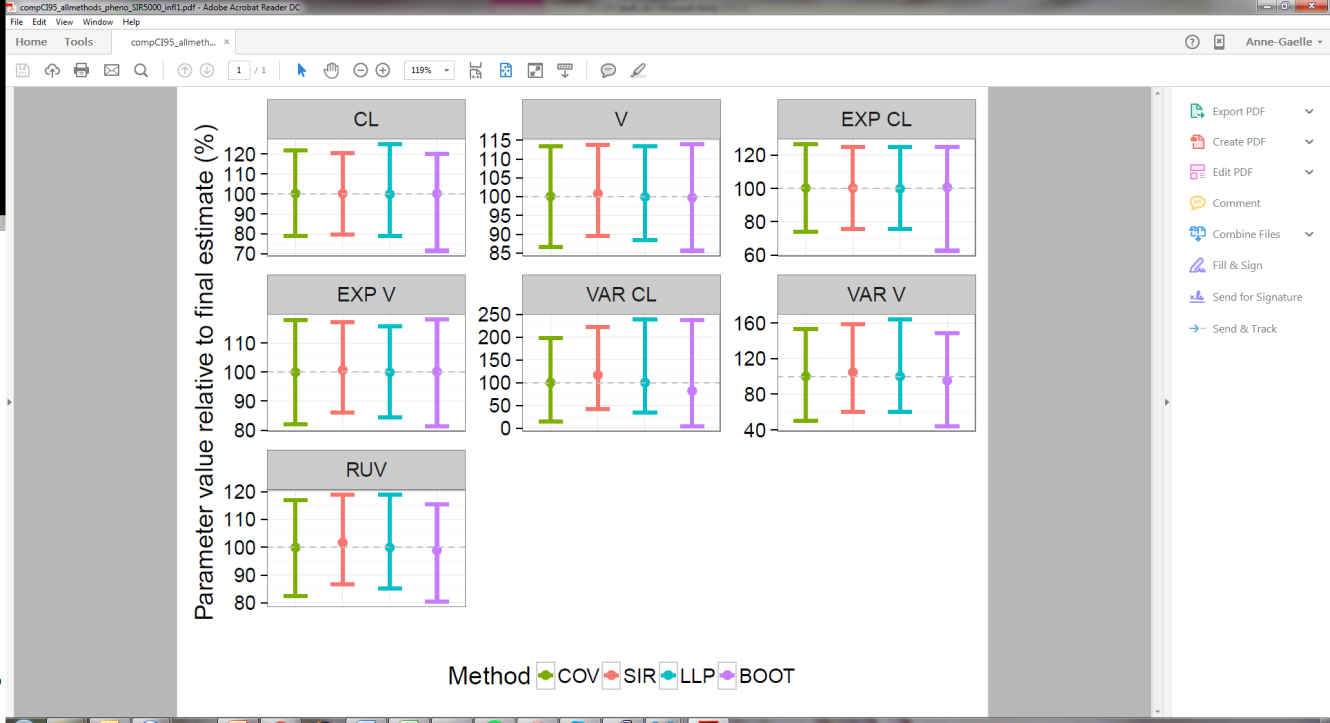 |
| --- | --- |

**Fig. A4**: Comparative 95% CI of the pefloxacin (panel a) and phenobarbital (panel b) model parameters between four uncertainty methods: covariance matrix (COV, green), sampling importance resampling (SIR, red), log-likelihood profiling (LLP, blue) and bootstrap (BOOT, violet). Vertical error bars represent the 95% CI and the points represent the median of the uncertainty distributions. All random effects are on the variance scale.
