# Supplementary material for: Improving the estimation of parameter uncertainty distributions in nonlinear mixed effects models using sampling importance resampling
Source: J Pharmacokinet Pharmacodyn. 2016 Oct 11;43(6):583–96. doi: 10.1007/s10928-016-9487-8 (PMC5110709; doi:10.1007/s10928-016-9487-8)
Supplement: Supplementary file 6 — Supplementary material 6 (DOCX 61 kb) [file 10928_2016_9487_MOESM6_ESM.docx]

**Online Resource 6: Changes in confidence intervals bounds and relative standard errors of the moxonidine model with increasing number of initial samples and inflation factor**

| Article title | Improving the Estimation of Parameter Uncertainty Distributions in Nonlinear Mixed Effects Models using Sampling Importance Resampling | |
| --- | --- | --- |
| Journal name | Journal of Pharmacokinetics and Pharmacodynamics | |
| Author names | Anne-Gaëlle Dosne^1^, Martin Bergstrand^1^, Kajsa Harling^1^, Mats O Karlsson^1^ | |
| Author affiliations | ^1^Department of Pharmaceutical Biosciences, Uppsala University, P.O. Box 591, 751 24 Uppsala, Sweden | |
| Corresponding author | Anne-Gaëlle Dosne: [annegaelle.dosne@farmbio.uu.se](mailto:annegaelle.dosne@farmbio.uu.se) | |
| 1. ***M/m* ratio**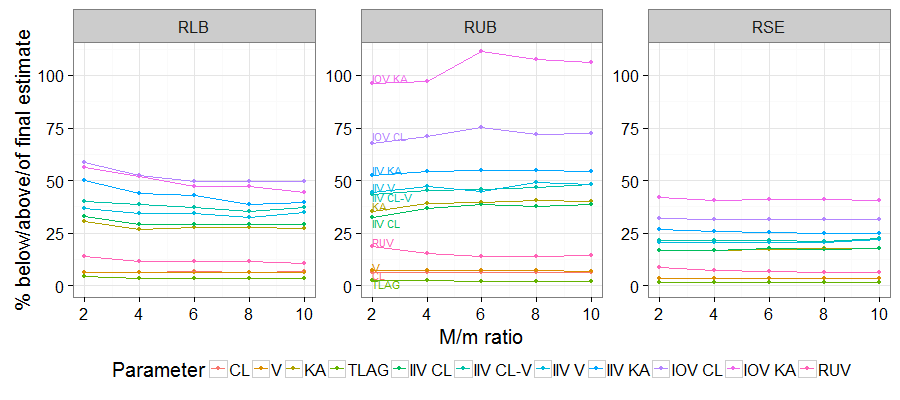 | |  |
| 1. **Inflation factor**   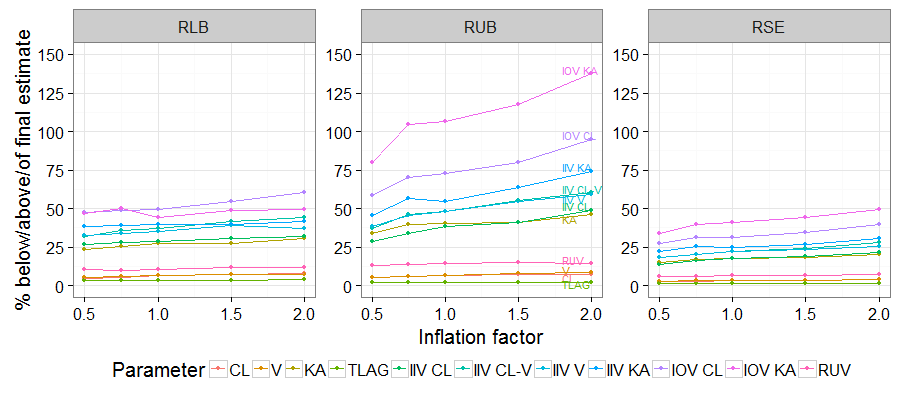 | |  |

**Fig. A6**: 95% CI bounds and RSE of the moxonidine model parameters over increasing number of samples (panel a) and inflation factor (panel b). RLB: relative lower bound, RUB: relative upper bound, RSE: relative standard error. SIR performed with 10,000 initial samples and without replacement. Y-axis interpretation: (a) After performing SIR with 2000 initial samples, the lower bound of the 95% CI of IOV KA (pink) is 56% below the final estimate, the upper bound is 96% above the finale estimate and the corresponding RSE is 41%. (b) After performing SIR with 0.5 deflation, the lower bound of the 95% CI of IOV KA (pink) is 47% below the final estimate, the upper bound is 80% above the final estimate and the corresponding RSE is 34%.
